# Biting midge dynamics and bluetongue transmission: a multiscale model linking catch data with climate and disease outbreaks

**DOI:** 10.1038/s41598-021-81096-9

**Published:** 2021-01-21

**Authors:** Tim W. R. Möhlmann, Matt J. Keeling, Uno Wennergren, Guido Favia, Inge Santman-Berends, Willem Takken, Constantianus J. M. Koenraadt, Samuel P. C. Brand

**Affiliations:** 1 Laboratory of Entomology, Wageningen University and Research, P. O. Box 16, 1700 AA Wageningen, The Netherlands; 2 IFM Theory and Modelling, Linköping University, 581 83 Linköping, Sweden; 3 School of Life Sciences, University of Warwick, Coventry, UK; 4 Zeeman Institute, University of Warwick, Coventry, UK; 5 Mathematics Institute, University of Warwick, Coventry, UK; 6 School of Biosciences and Veterinary Medicine, University of Camerino, 62032 Camerino, Italy; 7 GD Animal Health, PO Box 9, 7400 AA Deventer, The Netherlands

**Keywords:** Ecological modelling, Computer modelling, Entomology

## Abstract

Bluetongue virus (BTV) serotype 8 has been circulating in Europe since a major outbreak occurred in 2006, causing economic losses to livestock farms. The unpredictability of the biting activity of midges that transmit BTV implies difficulty in computing accurate transmission models. This study uniquely integrates field collections of midges at a range of European latitudes (in Sweden, The Netherlands, and Italy), with a multi-scale modelling approach. We inferred the environmental factors that influence the dynamics of midge catching, and then directly linked predicted midge catches to BTV transmission dynamics. Catch predictions were linked to the observed prevalence amongst sentinel cattle during the 2007 BTV outbreak in The Netherlands using a dynamic transmission model. We were able to directly infer a scaling parameter between daily midge catch predictions and the true biting rate per cow per day. Compared to biting rate per cow per day the scaling parameter was around 50% of 24 h midge catches with traps. Extending the estimated biting rate across Europe, for different seasons and years, indicated that whilst intensity of transmission is expected to vary widely from herd to herd, around 95% of naïve herds in western Europe have been at risk of sustained transmission over the last 15 years.

## Introduction

*Culicoides* (Diptera: Ceratopogonidae) biting midges transmit a wide range of pathogens of veterinary importance worldwide including Akabane virus, bovine ephemeral fever virus, Schmallenberg virus, African horse sickness virus, epizootic haemorrhagic disease virus, and bluetongue virus (BTV)^1–3^. Historically, BTV was not endemic in Europe but there were sporadic incursions into the continent. However, in the past two decades various serotypes of BTV invaded southern Europe^4^. The outbreak of BTV serotype 8 near Maastricht, The Netherlands, in 2006 was the first BTV outbreak ever observed above latitude 50^o^N anywhere in the world^5,6^. The virus subsequently demonstrated the capacity to subsist throughout the winter period in north-western Europe, re-appearing amongst commercial livestock in 2007^7^. Despite millions of vaccinations^8^, BTV serotype 8 re-emerged in France in 2015^9^, whilst other BTV serotypes remained circulating in southern Europe and caused periodic epizootic outbreaks in eastern European countries^10^.

BTV causes economic losses in terms of livestock morbidity and mortality, the cost of surveillance and vaccination, as well as indirect costs caused by livestock movement restrictions that lead to agricultural business interruption^11^. The persistence of BTV in Europe over the last two decades demonstrates the need to gain insight into the ecology of potential midge vector species across Europe as an aid to predict BTV transmission. Multiple biting midge species in Europe are known, or suspected to be, competent BTV vectors: species in the Obsoletus group^12–14^, *C. imicola* Kieffer, 1913^15^ *C. pulicaris* (Linnaeus 1758)^16^, and *C. punctatus* (Meigen, 1804)^17^.

Understanding the environmental factors that influence midge biting rates could result in more accurate predictions of BTV transmission, both within and between herds. Uncertainties about the biting behaviour of these midge species can be addressed by extensive field trapping and statistical analysis of trap catches. However, there is no consensus in the scientific literature on the most informative statistical method for analysing midge catch data. Published analyses of midge catch data include: discriminant analysis of seasonal midge abundance^18–20^, logistic regression of midge occurrence/absence^21^, linear regression on log-maximum midge catches^22^, mixed effect Poisson regression on daily midge catches^23,24^, as well as analyses combining such statistical methods^25,26^. Moreover, linking the outcomes of midge catch studies to a meaningful estimate of actual biting behaviour on commercial livestock has proven problematic. Midge biting rates for use in epidemiological modelling of BTV are typically estimated either by assuming some relationship to the true midge population size^27,28^, or by assuming a relationship directly to the vector to host ratio^29^. However, recent comparative studies of different methods of assessing midges have found significant variation in catch quantities depending on the method used, e.g. between collection by aspiration directly from a host compared to using black light traps^30^, or sweep netting compared to black light attraction^31^. Therefore, estimates of midge biting intensity will rely heavily on the catch method and the location of the catching, e.g. animal or a trap near the barn. Understanding the link between midge surveillance and biting intensity has been identified as a critical knowledge gap in understanding midge-borne disease transmission^32,33^.

**Figure 1:**
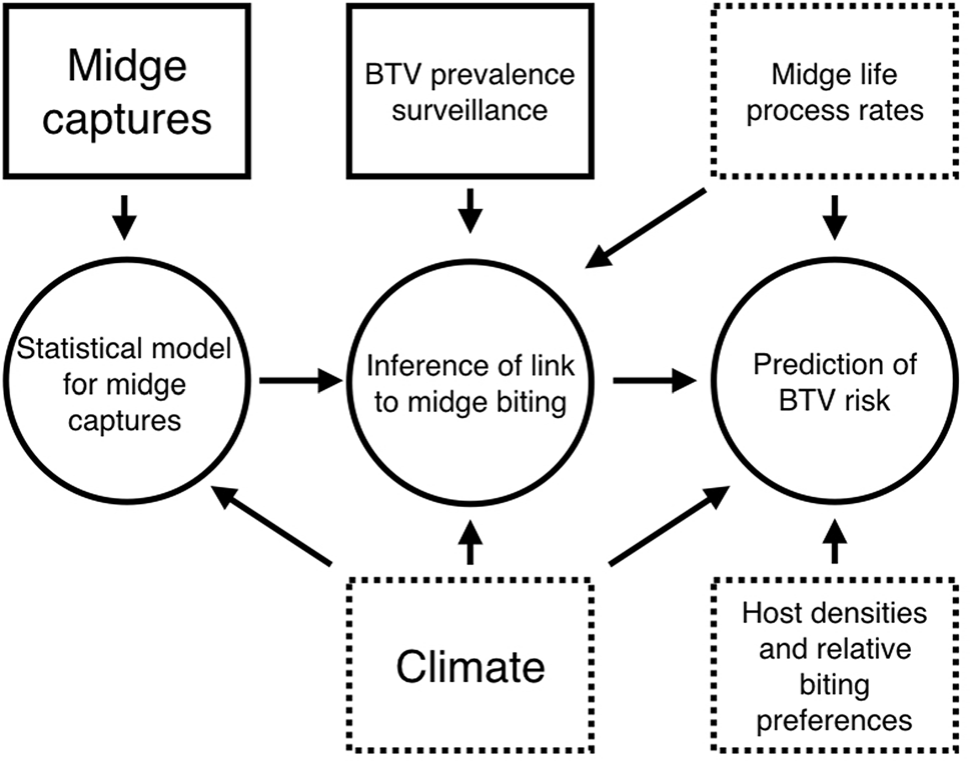
Schematic overview of the data and modelling workflow. Rectangular boxes represent data sources (solid borders denote data collected for the purpose of this study, dotted borders denote data available from literature or open-access digital archives). Circles represent models developed in this study, which have been either inferred from, or parametrised by, these data sources. Arrows denote dependency in model inference.

The goal of this study is twofold: first, to establish a link between the expected number of midges trapped in 24 h, to the expected number of bites received by a single cow over the same period. Second, to incorporate this link between catch number and biting rates into predictions of BTV risk across Europe in the recent past, focusing on within herd transmission. To achieve these goals we proceeded in four phases (see Fig. 1 for the workflow of this study), each of which depended on the outcome of the previous phases: (i) we made a series of field collections of midges using 24 h of trapping at a range of latitudes, habitats, and days of the year, (ii) we performed a regression analysis for the climatic, seasonal, and environmental factors that influence the daily catching of known European BTV vectors, (iii) we inferred a scaling parameter between the midge catch regression model and the true midge biting rate using a comparison between BTV surveillance of sentinel cattle in The Netherlands in 2007 and the corresponding daily midge catch number predictions, (iv) we used the estimated true biting rate to map BTV risk across Europe over the last 15 years.

## Results

### Midge catch data and regression analysis

#### Midge catch data

As presented in Möhlmann et al.^34^ a total of 50,729 *Culicoides* specimens (97.2% female, 1.3% male, 1.5% unidentifiable) were collected across 1329 separate trap collections between July 2014 and July 2015 at a variety of locations, representing farm (429/1329 collections), wetland (451/1329 collections) and peri-urban habitats (449/1329 collections), and in three countries: Sweden (410/1329 collections), The Netherlands (467/1329 collections), and Italy (452/1329 collections) using Onderstepoort black light traps (see Methods for further details of the collection protocol). These data were used as input for the modelling analyses presented here. From the collected midges, a total of 45 different species were identified^34^. The majority of the collections belong to the group of midge species known to vector BTV: the Obsoletus group consisting of *C. chiopterus* (Meigen, 1830)*, C. dewulfi* Goetghebuer, 1936*, C. montanus* Shakirzjanova, 1962*, C. obsoletus s.s.* (Meigen, 1818), and *C. scoticus* Downes & Kettle, 1952 (88.7%), followed by *C. punctatus* (2.3%) and *C. pulicaris* (2.2%). In total 46,697 of the 50,729 caught midges (92%) were known BTV vector species (Table 1). Another known European vector for BTV, *C. imicola*, was not caught during the study of Möhlmann et al.^34^ and therefore this species was not included in the analyses. The lowest numbers of midges known to transmit BTV were collected in Sweden (2964/46,697, 6.3%), followed by The Netherlands (10,359/46,697, 22.2%) and the highest numbers were found in Italy (33,374/46,697, 71.5%). In all countries, abundance was highest in farm habitats. Farm-associated species of *Culicoides* biting midges dominated the catch counts, not only in farm habitats where livestock hosts were present, but also in the other habitat types. This is consistent with findings in other midge catch studies using different trap types (e.g. Rothamsted suction traps^23^).

**Table 1:** Known midge vectors for BTV.

Midge species	Sweden			The Netherlands			Italy			Total
	Farms	Peri-urban	Wetlands	Farms	Peri-urban	Wetlands	Farms	Peri-urban	Wetlands	
Obsoletus group	1760	10	136	7865	43	1448	33,069	54	25	44,410
*C. pulicaris*	892	1	6	11	0	18	184	1	0	1113
*C. punctatus*	120	3	36	191	7	776	38	2	1	1174
Total	2772	14	178	8067	50	2242	33,291	57	26	46,697

Number of midges trapped for the known vectors of BTV in Europe, for each country (Sweden, The Netherlands, Italy) and habitat type (farms, peri-urban, wetlands). Data derived from Möhlmann et al.^34^.

#### Regression model for trap catches

We used a generalised linear mixed-effects model (GLMM) to explain the aggregated daily catches of all midge species associated with BTV transmission: the Obsoletus group*, C. punctatus,* and *C. pulicaris*. We considered combinations of predictor variables from three groupings of data: (i) climate data, as described in Methods, with mean values and coefficients of variation (CoV) (i.e. the ratio of standard deviation to mean values) over 24 h, 7 days and 30 days before collection, (ii) habitat categories "farm", "peri-urban", and "wetland", and (iii) a seasonality effect associated with collection time *t* included by considering annual, bi-annual, and tri-annual periodic sine and cosine functions on *t*. Predictor variables were associated with particular trap locations and collection times as fixed and/or random effects. We also included interaction terms between habitat and climate variables and between seasonality/habitat and country. See supplementary information for a complete list of model variables considered in the analysis.

**Figure 2:**
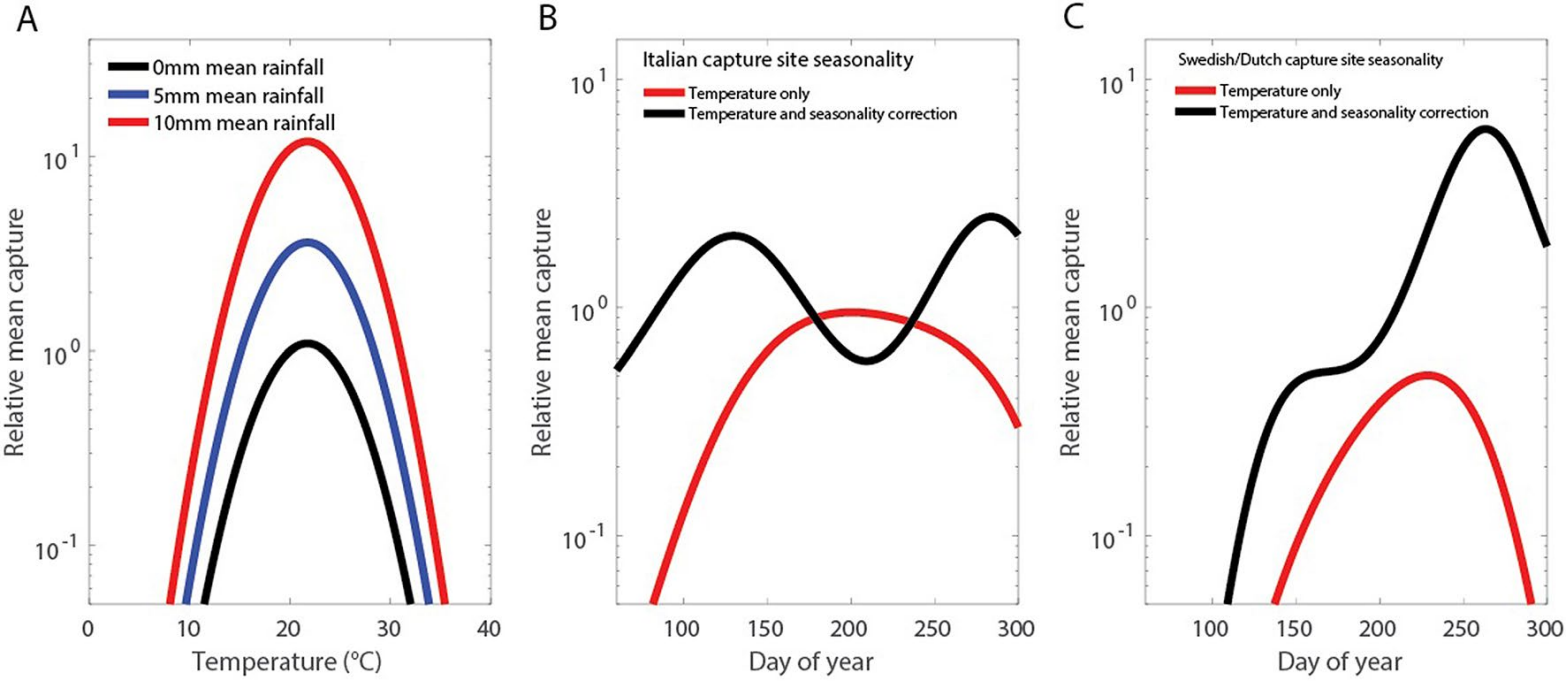
Expected midge catch size. Relative effect on midge catch size of varying mean daily temperature, mean precipitation over previous week, and catch day of year (in each case predictor variables being fixed). *Left* (**A**): Relative expected catch sizes for a range of temperatures and mean precipitation. *Middle* (**B**): Smoothed seasonal variation at the Italian sampling site if driven by observed local temperatures only (red curve) and with sine and cosine seasonality correction (black curve). *Right* (**C**): Smoothed seasonal variation at the Swedish and Dutch sampling sites if driven by observed local temperatures only (red curve) and with sine and cosine seasonality correction (black curve).

Potential predictor variables were eliminated in a stepwise process to find the best regression model for explaining the midge catch data (chosen using lower values of corrected Akaike information criterion (AICc) as the selection criterion; see Methods and supplementary information). The selected climate variables where higher values predicted greater numbers of midges catches were: the mean temperature over 24 h before catch collection (*P* < 10^–7^) and the mean daily precipitation over the week before catch collection (*P* < 10^–3^). Coefficient of variation for daily precipitation over the 30 days prior to collection was a significant predictor for larger catch size (*P* < 10^–10^), but only as an interaction term with wetland location. Climate variables where higher values predicted lower numbers of midge catches were: the squared mean temperature (*P* < 10^–4^) and mean wind velocity (*P* < 0.05) 24 h before collection. The squared temperature as negative predictor indicated that the activity of the European midge vectors of BTV considered in this paper was maximised at an optimal temperature of 20–21 °C (Fig. 2A). Significantly higher counts of caught midges were found at catch locations in farm habitats when compared to wetland or peri-urban habitats (Table 1, *P* < 10^–10^). The midge catch regression model was significantly improved by allowing the seasonal dynamics (driven by sine and cosine functions on time) of midges in Italy to be different from Sweden and The Netherlands. The midge seasonality at the Italian sampling locations could be summarised by using only annual-period predictors, whereas the seasonality observed in Swedish and Dutch traps was more complex involving bi- and tri-annual periodicity (Table S1).

The seasonality in midge catches could not be explained by seasonal variation in temperature alone. The best regression model included periodic predictor variables (sine and cosine functions on *t*) in conjunction with temperature and precipitation effects to predict the seasonality in midge catching (AICc improvement: $\Delta$AICc = 102.4 for model without periodic predictor variables). If periodic predictor variables were not included in the set of potential predictors, then the regression model could only predict uni-modal peaks in midge catch abundance, mid-July for Italy and mid-August for Sweden and The Netherlands (Fig. 2B,C; regression predictions smoothed using polynomial regression to investigate trends). However, the best regression model, including both periodic predictor variables and climatic variables, predicts bi-modal peaks in expected catches at the Italian site (peaks at early-May and early-October; Fig. 2B) and an irregular "humped" peak midge catch abundance for the Swedish and Dutch catch locations (peak late September; Fig. 2C). It should be noted that, as trapping was only performed from March to November, we only present statistical fits valid for the period between those months. Besides seasonality, we considered three groups of random effects in our analysis:

1. Particular sampling locations; this random effect group models variation in the regression coefficients between catch locations.
2. A daily varying spatial autocorrelation effect between all catch results in the same country collected on the same day; this random effect models wide-scale but short-term unobserved influences that affect all trap locations in nearby geographic areas.
3. Daily varying overdispersion between individual catches; this accounts for overdispersion in the midge catch distribution compared to the canonical Poisson distribution for count data.

**Figure 3:**
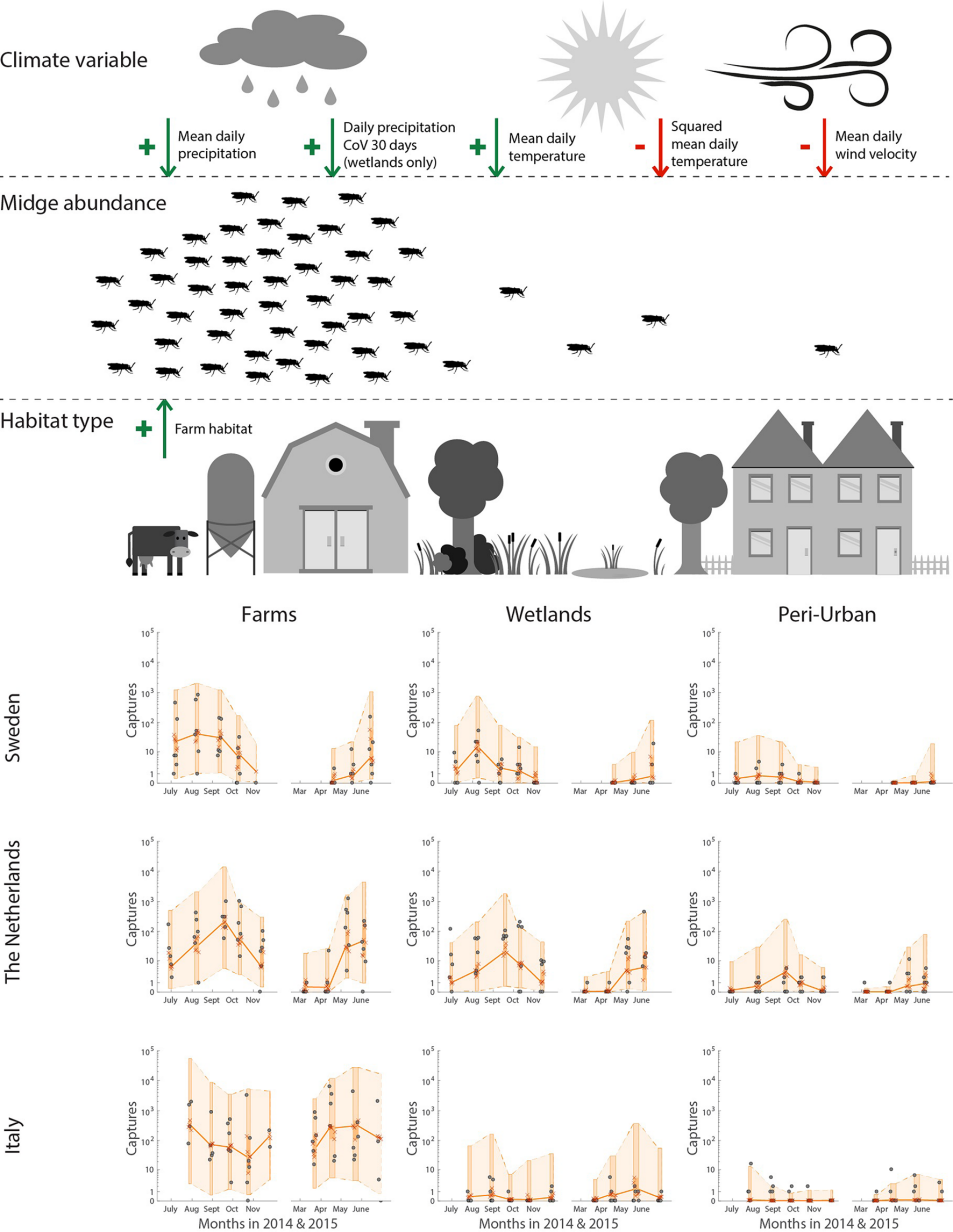
Diagram for midge activity model (top) with midge catch counts and catch model predictions (bottom). Black circles show catch counts and orange crosses are the median prediction of the GLMM over all possible random effects, with orange lines connecting the median predictions of catch weeks. Background shading show GLMM catch predictions between 5 and 95% percentiles for random effect coefficients, with deeper shading indicating a collection week. Images created by Viola Visser.

In the best regression model, there are location-group random effects for the intercept (baseline abundance at locations), the response to mean daily temperature, and the response to mean wind velocity over dawn and dusk before collection (see Fig. 3 for a schematic diagram of the catch model and predictions with random effect uncertainty). The location-group random effect on the intercept has a higher inferred variance than the overdispersion effect, indicating that the variability in midge catches between different sampling locations will typically be higher than the daily variability in repeating midge catches at one sampling location. The location-group random effects were negatively correlated amongst themselves, hence we would expect a sampling location with larger than typical baseline abundance to have a smaller than typical response to changes in temperature or wind velocity.

### Linking midge catch prediction to midge biting prediction

In the winter of 2006/2007, during the BTV transmission-free period, the Dutch government decided to establish a sentinel network of cattle herds to monitor the re-emergence of BTV-8 in 2007 using repeated milk ELISA testing^35,36^.

Repeated simulation of the percentage of cattle with virus detectable by milk ELISA tests in the sentinel herd network provided us with an inferred maximum likelihood estimator for the trap-to-bite scaling parameter. The average likelihood over many repeated simulations corresponded to a Monte Carlo estimate of the true marginal likelihood of the parameter $\xi$. Estimating the likelihood over a range of values of $\xi$ allowed the construction of a log-likelihood profile.

The maximum likelihood estimator (with a 95% confidence region) derived from the likelihood profile was ${\xi }^{*}=0.53 [0.40, 0.68]$ (Fig. S2). This estimator implies that 24 h of biting midge collection using the Onderstepoort blacklight trap, catches about double the number of biting midges (from the Obsoletus group, *C. pulicaris,* and *C. punctatus)* compared to the number of expected midges to bite a single cow per day.

### Bluetongue virus risk in European cattle and sheep livestock herds 2000–2016

We mapped BTV risk in Europe at the resolution of the E-OBS climate data set (0.25° lat./long. grid cells; see Methods). The risk metric used in this paper was calibrated using cattle serological data for BTV-8 serotype. Moreover, our midge capture data that did not include *C. imicola*. Therefore, we caveat that our results are most relevant to areas of Europe which have suffered from BTV-8 transmission vectored predominantly by midges from the Obsoletus species group, *C. pulicaris* and *C. punctatus*. Nonetheless, we give pan-European results for completeness of BTV transmission risk by these groups of biting midges.

The midge biting rate inferred in this study implies variation between the biting rates of herds, even when they experience the same climate. Therefore, we broadly categorise herds depending on their intrinsic level of risk: “low-risk” herds are bitten at a rate at the 5% percentile of the distribution of biting rates we would expect at their grid cell on each day, “median-risk” herds receive the 50% percentile of biting and “high-risk” herds receive the 95% percentile of biting. We denote the *p*^th^ percentile herd reproductive ratio ${R}_{p}$. We also calculated, for each day from 2000 to 2016 and each grid cell, the proportion of herds that we expected would have a locally increasing outbreak of BTV upon introduction ($P$). Where $P$ is the percentage of herds in the grid cell we expect to have a herd reproductive ratio greater than one.

**Figure 4:**
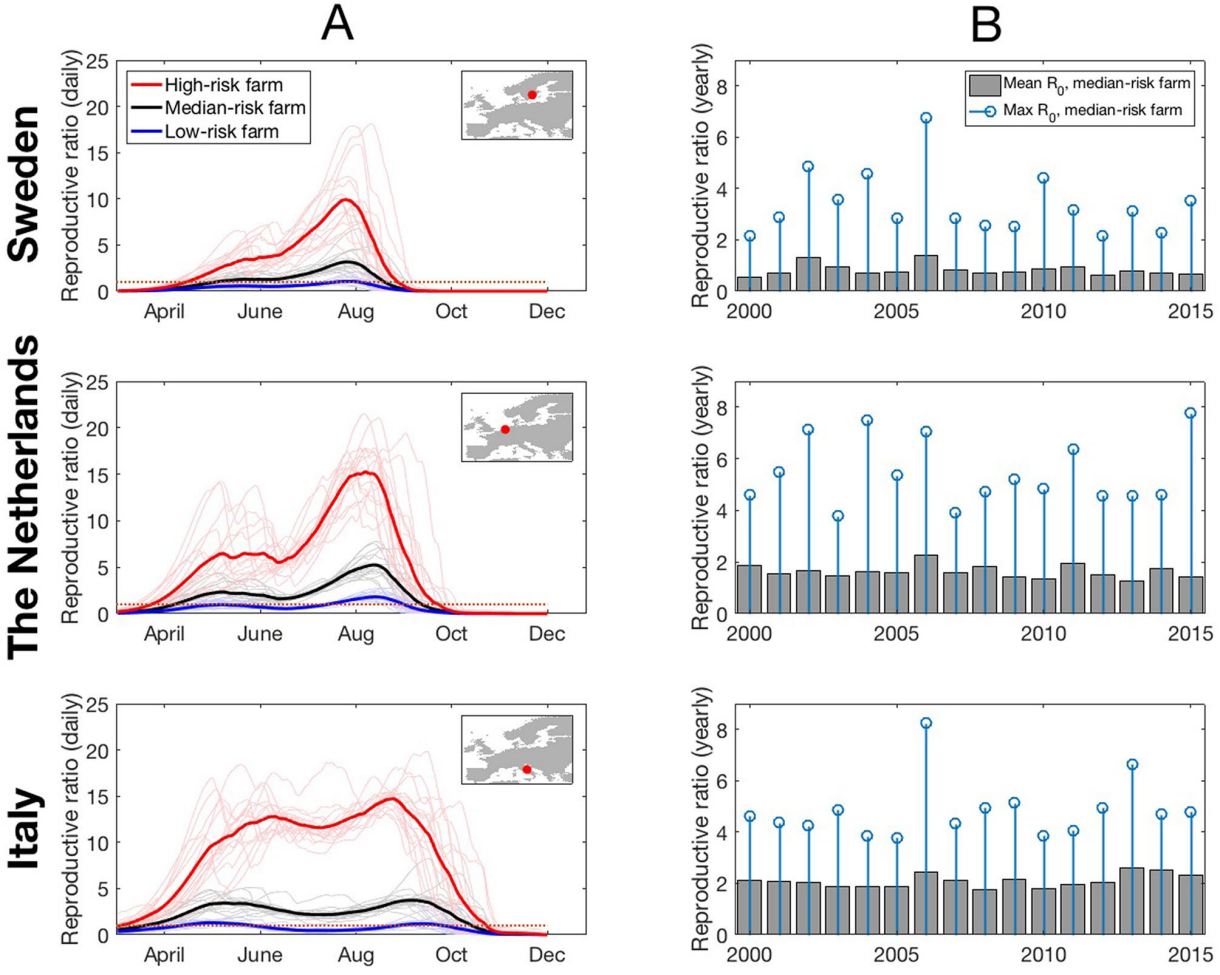
Predicted daily ${R}_{p}$ dynamics for farms in the area of the trap sites in each country (shown in insets). Left column (**A**): ${{\varvec{R}}}_{{\varvec{p}}}$ on each day of year (March-November) for *P* = 5% (blue curve), median *P* = 50% (black curve) and *P* = 95% farms (red curve) averaged over each year’s prediction for that day. Individual years (2000–2015) are shown as fainter curves of same colour. Right column (**B**): The mean ${{\varvec{R}}}_{50}$ over all days March-November for each year 2000–2015 (grey bars) with the maximum ${{\varvec{R}}}_{50}$ value, over all days March-November for each year 2000–2015 (blue circles).

Concentrating first on the grid cells that contained the sampling locations, the Swedish and Dutch sampling locations showed a notable peak in BTV reproductive ratio occurring around late July to early August for each risk level (typical peak reproductive ratio was 3.6 in Sweden and 5.3 in The Netherlands for median-risk herds). In contrast, the Italian sampling locations are predicted to have consistently fairly high BTV reproductive ratios from May to September (typical range was 2.1–3.8 for median risk herds) although with less distinct peaks in risk between late May and early September compared to the Dutch and Swedish locations (Fig. 4). At the Swedish sampling locations, median-risk herds are on the borderline for persistence during the early season (May–June;${1< R}_{50}<1.5$) and have only a short period (late July-early August) during which BTV is expected to multiply (i.e. ${R}_{50}>1.5$). On the other hand, it was predicted that a high-risk herd in the grid cell containing the Swedish sampling locations would be at risk of a serious outbreak throughout May to August. In the grid cell containing the Dutch sampling sites, high-risk farms will also have a significantly longer transmission season and greatly elevated transmission intensity compared to median-risk herds (Fig. 4A). Each year, the March-November daily mean for ${R}_{50}$ decreased towards latitudes that are more northern. However, this trend was not observed for peak ${R}_{50}$, where peak values for The Netherlands could exceed those in Italy (Fig. 4B). Consistent with other retrospective analyses of BTV risk, we found that 2006 was an outlier year for mean ${R}_{50}$.

**Figure 5:**
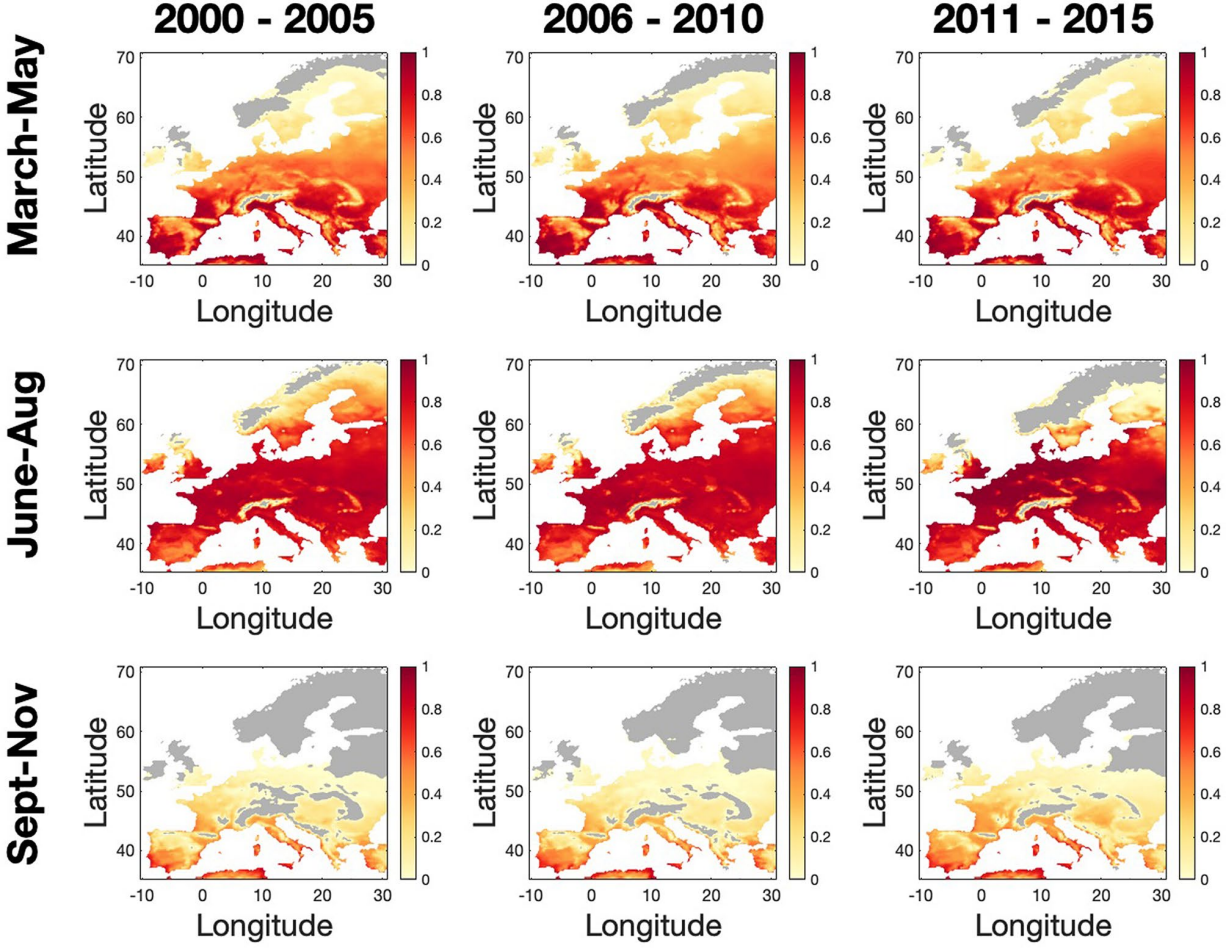
Estimated proportion of naive herds at risk of BTV outbreaks ($P$). Estimations over time periods of five years and early (March–May), mid (June–August) and late (September–November) seasons mapped across Europe for 2000–2015 (spatial cell scale = 0.25° lat./long.). Colours indicate increasing proportion of risk from yellow to red (zero risk is coloured grey). It should be noted that the risk map was calibrated using serological data for BTV-8 serotype and midge capture data that did not include *C. imicola* (see Results and Discussion).

Our results for $P$ fluctuated daily (see supplementary Video 1), therefore we present seasonal averages (Fig. 5). The seasonal average of $P$ for a grid cell can be interpreted as the chance that an infectious animal imported into a local herd in the season, will be introduced into a herd where an outbreak can occur. During the early months of the BTV transmission season (March–May; top row Fig. 5) we found that a large proportion of herds (> 75–95%) are already estimated as capable of sustaining BTV outbreaks in a number of regions including: southern Europe (southwest Spain and Portugal, most of mainland Italy), eastern Europe (Hungary, Bulgaria, Romania), southwest France, and the Mediterranean coastal regions of Algeria and Tunisia and western Turkey (Fig. 5). In western Europe (northern France, Germany, and The Netherlands) and in the Baltic States more than half of herds are expected to be already capable of sustaining BTV transmission in these early months. For the most recent five years (2011–2015), there is a notable increase in the percentage of farms capable of sustaining BTV transmission in the Ukraine and Belarus during the early season compared to 2000–2005. This contrasts with findings in Ireland, UK, Denmark, Sweden, where we find that the percentage of herds capable of sustained BTV transmission in March–May was low in the years 2000–2005 (< 25–35%) and this has generally remained unchanged by 2011–2015 (Fig. 5).

During the mid-season (June–August) we estimated that BTV transmission could be sustained at more than 80% of herds throughout mainland Europe south of 58^**o**^N, apart from mountainous regions (for example the Alps, Pyrenees, and Carpathians) and in southern/central Spain. More than 70% of herds were expected to sustain BTV transmission in the midland and southern regions of England and parts of southern Sweden, but we expected lower percentages of herds being capable of sustaining transmission in Norway, Finland, Scotland, Wales, northern Ireland and Ireland. In the last five years considered (2011–2015) the proportion of farms expected to sustain BTV transmission was higher in mainland western Europe (46^**o**^N-53^**o**^N) during the summer compared to 2000–2010. In this most recent period, 95–100% of herds are predicted as being at risk of sustained transmission. In fact, our model predicted that on mainland Europe, BTV risk during the mid-season was higher in the north than further south over the last five years as ideal climatic conditions for BTV transmission (see Brand and Keeling^37^) are more frequently realised. At the tail end of the transmission season (September–November) less than half of the herds across Europe were expected to be able to sustain a BTV outbreak, apart from southwest Spain and Portugal and some coastal regions elsewhere in southern Europe (Fig. 5).

## Discussion

By combining multi-scale synthesis of field data and modelling, we connect findings at the scale of individual midge catches in traps to BTV seroprevalence amongst herds of cattle, leading to herd-level risk assessments at the pan-European scale.

Midge catch studies based on trap catches in a specific country, region, or habitat are reasonably common in the literature. Rather than focusing on midge catches in one country, we spread our trapping effort over a range of different latitudes, thereby observing the activity of BTV vectors under a wider range of climatic conditions. This improves confidence in our regression model, in particular in identifying an optimal temperature for the activity for the BTV vectors found in this study (20–21 °C). Trapping studies that focused on warmer southern European countries only, have found that higher temperatures implied a lower probability of catching midges of the Obsoletus group^38^. We would conclude that this was due to trapping whilst temperatures were higher than optimal. This makes extending conclusions about BTV transmitted by species from the Obsoletus group based on trapping conducted in a warm climate to north-western Europe unreliable (e.g. Guis et al.^29^). On the other hand, in this study we did not find any *C. imicola*, the predominant vector of BTV in southern Europe, most likely because our study sites were outside the distribution range of *C. imicola*^39^. Whilst we extend our predictions to southern Europe for completeness, we acknowledge that our conclusions about BTV risk should be treated with caution in areas where *C. imicola* are abundant, such as large areas of Spain, western Italy, and Mediterranean islands. BTV risk is likely to be underestimated for these regions in our model, as *C. imicola* is thought to be responsible for 90% of the transmission in those areas^37^.

In addition to the identification of an optimal temperature for biting midge activity, we found that larger variation in daily precipitation over the 30 days prior to collection resulted in larger catch size in wetland locations. This precipitation variation effect on midge catches has been detected previously^38^. A possible explanation is that higher precipitation variation is correlated with greater availability of unflooded larval development sites (see discussion in Calvete et al.^38^). Our observation that this effect occurs only in wetland habitats seems to agree with this argument.

A novel feature of the modelling conducted in this study is that it incorporates real field data for both midge catches and transmission to cattle hosts. We were able to infer a proportionality factor between the rate of catching midges and their biting rate from a seroprevalence time series^33^ using maximum likelihood for dynamical system inference. This answers a major challenge in the field of vector-borne disease epidemiology by rigorously connecting surveillance to vector biting (see the discussion in Mullens et al.^33^). We found that the biting rate per day was expected to be about 50% of the prediction of 24 h of trap collection. This is a surprising result in the light of a study where midges were collected by aspiration, that found livestock to be much more attractive to midges than the Onderstepoort black light trap that we used for midge collection in our study^30^. However, it should be noted that in the aspiration study, nearly all midges were collected from animals during dusk, and collection ceased whilst the rate of trap catches was still rising. The trap-to-biting ratio should be interpreted with some caution since it is inferring the best "effective" biting rate proportional to our catch predictions; that is, the rate of bites that will cause infection. Therefore, errors in estimates drawn from the literature will lead to errors in inference. For example, if the BTV vector competences used in this study are an underestimate for Dutch midges transmitting BTV-8 then the inferred biting rate to catch ratio will be an overestimate to best fit to the observed cattle BTV-8 prevalence. In the BTV literature the effect of uncertainty in midge bionomic rates on predictions of BTV transmission have been investigated using sensitivity analysis (e.g. Gubbins et al.^27^). We did not perform a sensitivity analysis because we inferred the best “effective” biting rate, and many of the transmission parameters occur as formally indistinguishable effects (such as the BTV vector competence example above). We emphasise that all the serological surveillance data used in this paper was for BTV-8, and extending our findings to midge-to-livestock transmission rates of other serotypes might leaded to biased results.

We estimate that the risk of BTV outbreaks amongst naive cattle herds has increased in the past five years. South, central, and eastern Europe are estimated to have been at increased risk. Indeed, there has been a succession of outbreaks of various serotypes of BTV between 2010 and 2015 in both eastern (Hungary, Slovenia, Romania, Croatia) and southern parts (Italy, Spain, southern France) of Europe^10^, as well as the re-emergence of BTV-8 in central France^9^. High temperatures in southern Europe might decrease BTV risk when conditions become too hot for effective transmission^37^. However, midge species adapted to these conditions (e.g. *C. imicola*) still pose a threat for BTV transmission in these areas. Moreover, we must emphasise that the reproductive ratio being above unity is a necessary, rather than sufficient, condition for local growth of cases. Therefore, the spatial mapping of BTV risk should be interpreted as the percentage of herds at risk of a multiplying BTV outbreak if BTV was introduced, rather than predicting when and where BTV cases would definitely occur. The actual distribution of cases observed year-on-year also depends upon livestock vaccination coverage, the birth rate of new BTV-naive livestock, and the introduction mechanism of BTV into herds. In particular, the invasion velocity of BTV has been closely investigated using statistical regression methods^40–42^. In this study, we have connected statistical regression methods for midge capture abundance to a mechanistic model of local BTV transmission within herds. A natural further step would be to combine the existing statistical work on BTV invasion velocities with the results in this study on local growth of BTV and a mechanistic model of BTV spatial spread. The theoretical connection between reproductive ratios and invasion velocities is now classical in the reaction-dispersal literature for both spatially homogeneous^43^, and inhomogeneous^44^, models. For example, in a simple susceptible-infected-recovered (SIR) model with diffusive movement of the population (diffusion constant *D*) the relationship between invasion velocity (*c*) and R_0_ is $c=\sqrt{{2D(R}_{0}-1)\gamma }$^55^. Even in this simple setting knowing the R_0_ value is insufficient to predict the invasion velocity. However, it is possible to create a theoretical relationship between BTV invasion velocity and the R_0_ modelling used in this paper. We consider this a very promising avenue of future research.

In this study, we predict that the reproductive ratio is likely to vary significantly between herds, even if the herds experience similar climatic conditions. We find that the 5% of herds least at-risk, and experiencing climatic conditions identical to those at our trap sites, will virtually never have a strongly growing BTV outbreak. On the other hand, the 5% of herds most at risk could sustain BTV epidemics at any point between March-November if there were sufficient naive cattle. This observation may explain why complete elimination of BTV from European livestock herds remains elusive, despite high vaccination coverage ending significant “travelling wave”-type BTV epidemics, as seen in France in 2007–2009^45^. Our prediction that BTV risk will vary strongly from herd to herd is based on statistical regression; we have not identified mechanistic causes of variable risk. Identifying the causes of variation in BTV risk between locations would be highly important for disease control. A more complete picture of BTV risk requires even more extensive synthesis of midge catches data across Europe, which is an area of on-going research effort^18^.

## Methods

We have used a multi-scale modelling approach combining multiple modelling and inference types and techniques: generalised linear mixed-effect models (GLMMs), a mechanistic BTV transmission simulation model, marginal maximum likelihood inference, and direct calculation of resultant BTV risk predictions. We have parameterised our models with data from multiple existing sources (spatio-temporal climate time series, livestock density estimates, midge life process rates, BTV incidence time series), as well as data from our recent field study on the activity of midges at different latitudes in Europe^34^. The workflow and data sources of this study are summarised in Fig. 1.

### Biting midge collection and identification

The method of biting midge sampling and identification was described in our earlier study^34^. In short, three habitat types were defined in which *Culicoides* specimens were collected: “farm”, “peri-urban”, and “wetland”. Traps were placed within a 50 m radius of cattle, a house, or waterbody, for farm, peri-urban, and wetland habitats respectively. Habitat types generally matched the classification of the CORINE European Land cover database^46^. Collections were performed in Sweden (surroundings of Linköping N58.410808, E15.621532), The Netherlands (surroundings of Wageningen N51.964795, E5.662898), and Italy (surroundings of San Benedetto del Tronto N42.949483, E13.878503). Onderstepoort Veterinary Institute black light traps were placed at three independent locations for each selected habitat type. Traps were at least 100 m apart to prevent overlap of the active trapping radius^47^. More details and exact trap locations can be found in Vogels et al.^48^ and Möhlmann et al.^34^. Collections were performed for six consecutive days in each month in all three countries, during the period from July 2014 to June 2015 except the winter months of December, January and February (and March for Sweden). Traps were emptied and repositioned at different locations every 24 h. Collections were sorted and stored in 70% ethanol at − 20 °C. Samples were identified to species level using the Interactive Identification Key for *Culicoides* (IIKC) developed by Mathieu et al.^49^.

### Predictor variables for midge activity

Tinytag® meteorological data loggers (Gemini Data Loggers, Chichester, UK) were used to record local temperature and relative humidity every 30 min during the collection period from 17th July 2014 until 3rd July 2015. For each habitat in each country, one data logger was used (3 countries × 3 habitats). Additional meteorological data was collected from a number of sources: hourly wind velocities and local temperatures were available from the weather station closest to the trap location in Sweden (Swedish Meteorological and Hydrological Institute (SMHI) Linköping weather station), The Netherlands (Koninklijk Nederlands Meteorologisch Instituut (KNMI) weather stations “De Bilt”, “Deelen”, “Cabauw”, and “Volkel”), and Italy (San Benedetto del Tronto weather station). Finally, additional minimum, maximum, and mean daily temperatures along with precipitation and air pressure were sourced from the E-OBS European climate database^50^. This data was available at a daily temporal resolution and a spatial resolution of 0.25 degrees latitude and longitude. For climatic variables such as wind only one source per area could be used, whereas for temperature we had the opportunity to explore the most predictive of a number of data sources per country. Habitat effect was included by using the habitat types (farm, peri-urban, wetland) as a categorical predictor in the regression models.

### BTV-seroprevalence survey data from Dutch sentinel herds

After the initial BTV outbreaks in 2006, the Dutch government decided to establish a sentinel network of dairy cattle herds in the winter of 2006/2007 to monitor the re-emergence of BTV-8 in 2007, using repeated milk ELISA testing^35,36^. For study purposes, The Netherlands was divided into 20 compartments based on geographic boundaries as proposed in Commission Decision 2005/393/EC. In each compartment, at least 10 randomly selected herds had to be sampled (with at least sixteen cows per herd) to obtain the required sample size. Herds were not necessarily completely BTV-8 seronegative at initial investigation, but cows designated for the sentinel program had to be BTV-8 seronegative at the moment of selection in May 2007. Therefore, dairy herds to act as sentinels for BTV incidence were selected, that had at least sixteen seronegative cows and at least 50 cattle in total. Monthly milk samples were collected from the sentinel cows in each herd unless prevalence had already reached 100%. The first round of monthly testing of sentinel cows was done in June 2007 and continued until January 2008. The monthly milk samples were tested at GD Animal Health, Deventer The Netherlands for antibodies to BTV-8 using a commercially available ELISA test. For further details on the sampling protocol and commercial ELISA see Santman-Berends et al.^35,36^.

### Regression model for biting midge catches

A preliminary inference investigation using a hurdle negative binomial model to explain trap catches, inferred using Metropolis–Hastings MCMC, suggested that the excess of zero catches could be explained without zero inflation using the hurdle mechanism. We therefore used GLMM regression for its convenience and flexibility. Regression for the fixed effect coefficients and variance parameters of the random effects was performed via maximum likelihood using the Laplace approximation method implemented by the **fitglme** MATLAB® function. We followed the recommendation guidelines of Bolker et al.^51^ for using generalized linear models in the context of applied ecology, starting from a model with a full set of predictors and performed systematic model reduction using AICc to score model improvement (backwards model selection). AICc is a well-established information criterion for model selection since it is easy to calculate and interpret for GLMMs. The AICc difference between two models (ΔAICc) estimates the relative likelihood of the two models (with $\sim {\text{exp(}} - \Delta {\text{AICc}}/2)$ as relative likelihood for the model with greater AICc). Moreover, the model selected by lowest AICc is also the model that would be selected by leave-one-out cross-validation^52^.

Candidate predictor variables for removal from the model were chosen by assessing which fixed-effect coefficients had the greatest P value (for the null hypothesis that the coefficient is zero) and which random effect coefficients had the smallest predicted standard deviation. This was followed by trialling the removal of either of the variables and removing the variable that reduced AICc the most, until no further reduction could be made. Several trials were made where we started from a number of different highly over-parameterised models, which all ended with the same best model.

For any given combination of predictor variables, the catches were assumed to be conditionally Poisson distributed, with the conditional mean for each collection defined by the log-link relationship,

1 $$ln\left( {\mu_{lct} } \right) = \beta \cdot X_{lt} + b_{l} \cdot Z_{lt} + \rho_{ct} + \varepsilon_{lt}$$

where $\beta$ is the fixed effect regression coefficient that applies to all catches, with ${X}_{lt}$ denoting the fixed effect predictors at trap location *l* on day *t*. ${b}_{l}\sim Normal(0,\Sigma )$ are the location-grouped random effect regression coefficients with covariance matrix $\Sigma$, with ${Z}_{lt}$ denoting the random effect predictors at location *l* on day *t*. The number of midges per catch was highly variable. Therefore, we included an independent random effect for each catch location and day ${\varepsilon }_{lt}\sim Normal(0,{\sigma }_{\varepsilon })$. This corresponds to assuming that the number of midges per catch is Poisson log-normally distributed (a standard distribution for over-dispersed count data^53^). Spatial autocorrelation has been found in other midge catch regression studies^38^, so we also included an autocorrelation random effect grouped by country of trapping and day; ${\rho }_{ct}\sim Normal(0,{\sigma }_{\varrho })$. This effect accounts for events influencing trap catch at a wider scale than just the location definition of each trap. See supplementary information for full model variables, regression coefficients and random effect variance estimates as well as AICc improvements from other models.

### Connecting the regression model for midge captures to a midge biting prediction for herds

We connected the regression model for daily midge trap catch to a prediction of daily biting on cattle. In the BTV modelling literature it is common to assume that the vector-to-host ratio, and therefore the biting rate per livestock host, is proportional to the expected capture rate of a midge trap catch regression model^28,54^. In this study, we assumed that the biting rate per livestock host is proportional to the regression model presented in Results, with the major difference that we infer the scaling parameter that best fitted the serological data from Dutch sentinel herds. The location-group random effects in the regression model modelled unexplained spatial variation in average midge capture counts between trapping locations. We assumed that this unexplained spatial variation in midge abundance (as measured by trapping) mirrored the spatial variation in midge biting between different herds. Combining the proportionality assumption with the spatial variation assumption gave the biting rate among herds as,

2 $${\text{Biting }}\,{\text{rate }}\,{\text{of }}\,{\text{susceptible}}\,{\text{ midges }}\,{\text{per}}\,{\text{ cattle }}\,{\text{at }}\,{\text{herd}}\,h\,{\text{on}}\,{\text{ day}}\,t = \xi \mu_{ht} .$$

where ${\mu }_{ht}$ is the expected trap catch from the regression model given the climate condition local to herd *h* on day *t* and $\xi$ is a scaling parameter between the mean catch prediction and the biting rate prediction. In order to infer the proportionality factor between the catch model and the daily biting rate ($\xi$), the outcomes of a dynamic transmission model for each herd were linked to the observed BTV seroprevalence data (see—“Mechanistic transmission model for BTV transmission within herds” section).

### Mechanistic transmission model for BTV transmission within herds

**Figure 6:**
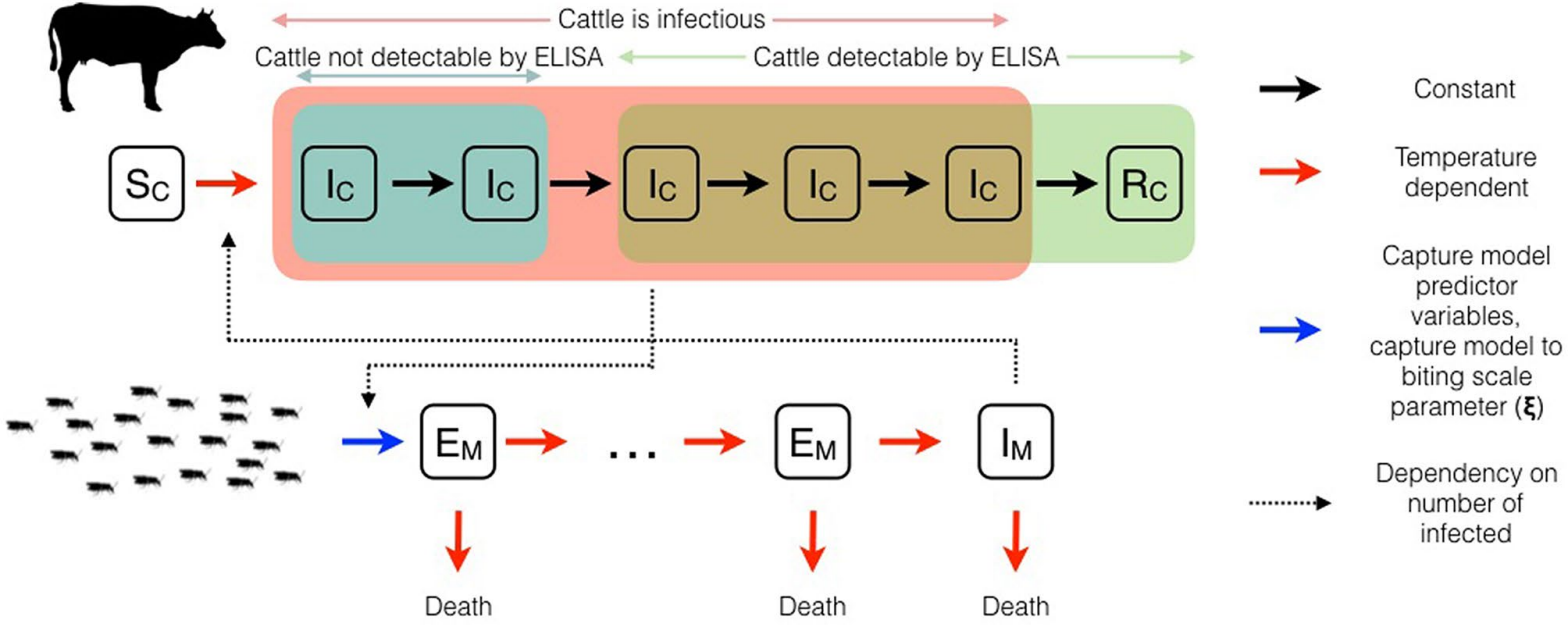
Schematic representation of the cattle herd level BTV transmission model. The population of cattle and infected biting midges are divided amongst discrete disease compartments. BTV latent midges (E_M_) enter the model at a rate proportional to the daily prediction of the catch model. The extrinsic incubation period for latent midges is modelled as a multi-stage process before midges become infectious (I_M_). Susceptible cattle (S_C_) become infectious cattle (I_C_) after a bite from infectious midges (I_M_), to become resistant cattle (R_C_) in time (also modelled as a multi-stage process; red box). Transitions are shown as solid lines, coloured according to their dependence on environmental variables: constant per-capita (black), daily mean temperature dependent (red), all predictor variables of capture model and the catch-to-bite scale parameter ${\varvec{\xi}}$ (blue). Dotted lines indicate where the number of infected individuals in one species increases the incidence rate in the other species. Outcomes of the model are linked to observed cattle milk serology time series by the first two infectious stages for cattle with virus being undetectable by ELISA (blue box), whereas subsequent infectious stages and the recovered stage are detectable by ELISA (green box). The likelihood function for $\xi$ was inferred by marginalisation over the latent stochastic variables affecting model outcomes (e.g. herd-specific random effects, daily fluctuations in midge activity). Used images were available under open licence Creative Commons Deed CC0.

We used a dynamic and mechanistic model of BTV transmission within herds, which was then matched to the data from the Dutch sentinel study. The dynamic BTV transmission model was formulated using disease compartments and rate-based transitions (see Keeling and Rohani^55^ for further details on this class of transmission model). In addition, it is in most respects similar to the model presented by Gubbins et al.^27^ in treating infectiousness amongst cattle, and latency amongst midges, as multi-stage processes that evolve deterministically. In particular, we follow Gubbins et al. in modelling the life processes of the infectious and latent midges (mortality, extrinsic incubation of BTV, and biting) as temperature dependent, and therefore varying daily with local background atmospheric temperature. (see Fig. 6 for a schematic diagram of the transmission model).

The dynamic and mechanistic BTV transmission model used in this study describes the evolution of the numbers of susceptible, infected, and recovered cattle as well as latent and infectious midges for each herd (Fig. 6). Temperature-dependent midge bionomic rates were used for biting frequency of individual infectious midges^56^, the incubation rate of BTV within the midge^57^, and for the midge mortality^58^. The midge bionomic rates at each herd on each day were determined by the local mean temperature day according to the E-OBS climate dataset (see above—*Predictor variables for midge activity*). We modelled the incubation period of BTV within the midge vector as a ten-stage process, which is within the range of best fit models found in meta-analysis and laboratory studies of BTV incubation^57^ (see supplementary information for complete model details and literature estimates for rates).

The period during which BTV-infected cattle are detectable using an ELISA test (typically from 8–9 days post-infection onwards^59^) does not match the period during which the cattle are infectious (rapidly post-infection and then for an average of 20.6 days^27^). BTV-infected cattle can be in four states that are relevant to transmission modelling and their milk serology: 1) uninfected and susceptible to BTV, 2) infectious but undetectable by milk ELISA, 3) infectious and detectable by milk ELISA or, 4) non-infectious recovered from BTV but still milk ELISA positive. The BTV infectious period for cattle is usually modelled as a 5-stage process^27^, therefore it was convenient to model cattle in the first two stages of their infectious period (an average duration of 8.2 days) as infectious but undetectable. Cattle in the final three stages of the BTV infectious period are infectious and detectable (see Fig. 6 for a schematic representation of the BTV transmission and serology model).

The rate at which new susceptible midges arrived to bite cattle was assumed to be proportional to the expected number of midges from the regression model (${\mu }_{ht})$, with predictions of midge catches on each day and in each regional compartment of The Netherlands whilst the sentinel herd study was ongoing using the E-OBS historic climate records (see above—“Connecting the regression model for midge captures to a midge biting prediction for herds” section). The random effects in the catch model imply that ${\mu }_{ht}$ is a daily varying random variable, and that our transmission model is in the class of piecewise-deterministic Markov processes^60^. We assumed that the location-grouped random effects observed in the catch model became herd-grouped random effects for the biting model. In other words, we assumed that the high variance in midge catching between trapping location reflects high variance in midge biting between different cattle herd locations. Although an assumption, this would explain the highly variable intensity of BTV transmission observed between different herds in The Netherlands sentinel survey despite each herd experiencing a similar climate^36^. Because the herd locations were known only as geographic compartment occupancy, the daily local climate variables from the gridded E-OBS data used for predicting ${\mu }_{ht}$ were averaged over all spatial grids overlapping the herd’s geographic compartment. Accessing detailed and spatio-temporally resolved wind data across Europe was challenging, therefore we used the long-term average wind velocity of The Netherlands weather stations (see above—“Predictor variables for biting midge activity” section) as a constant predictor.

When simulating an outbreak of BTV within a herd, we first determined all relevant climatic predictors for the herd's regional compartment and the daily temperature dependent midge bionomic rates. Second, we generated the herd-grouped and daily varying random effect coefficients which determined how biting at the herd from susceptible midges varied from a median prediction. Third, we solved the resultant deterministic BTV transmission model for each farm using the **ode45** MATLAB® function (see Fig. 6 for an overview).

### Inferring the catch-to-biting scale parameter from serological data

We inferred a maximum likelihood estimator for the trap-to-bite scale parameter by repeated simulation of the percentage of cattle detectable by milk ELISA tests herds in the sentinel herd network. For this we used the climatic conditions of The Netherlands in 2007**,** and at each simulation repeated redrawing the unobserved random effects for each herd and day. The average likelihood over many repeated simulations corresponds to a Monte Carlo estimate of the true marginal likelihood of the parameter $\xi$. Estimating the likelihood over a range of values of $\xi$ allowed the construction of a log-likelihood profile.

The stochastic elements of the piecewise-deterministic BTV transmission model were (i) the herd-grouped random coefficients (this modelled how biting varied from herd-to-herd) and (ii) the daily varying random effects (this modelled how biting varied from day-to-day). It is convenient to denote ${W}_{h}= ({{b}_{l}}^{(h)},{{\rho }_{0}}^{(h)},{{\rho }_{1}}^{(h)},{{\rho }_{2}}^{(h)},...,{{\epsilon }_{0}}^{(h)},{{\epsilon }_{1}}^{(h)},{{\epsilon }_{2}}^{(h)},...)$ as the collection of all stochastic elements for herd *h*. For each simulation of the transmission model in each herd, we first drew ${W}_{h}$ from their inferred distribution (see supplementary information for distribution parameters of best fitting model).

The likelihood of ${W}_{h}$ and $\xi$ for each herd *h* was the chance of selecting the numbers of ELISA seroconverted cattle observed at the herd each month by The Netherlands sentinel study from the underlying distribution of ELISA detectable cattle implied by simulating the transmission model conditional on $(\xi ,{W}_{h})$,

3 $$L_{h} \left( {\xi ,W_{h} } \right) = P({\text{Serology }}\,{\text{data }}\,{\text{collected }}\,{\text{at }}\,{\text{herd }}\,h| \xi ,W_{h} ).$$

Since we were not interested in inferring the specific values of ${W}_{h}$ for each herd, they were treated as “nuisance” parameters. We inferred a maximum likelihood estimate, with confidence intervals, for $\xi$ by first estimating the marginal likelihood for $\xi$ (that is the likelihood after integrating over all possible values of the nuisance parameters) at each herd *h*,

4 $${L}_{h}(\xi ) = \int {L}_{h}\left(\xi ,w\right)f\left(w\right)dw.$$

where $f$ is the density function for the distribution of random effects derived from the trapping model. The marginal log-likelihood function for the trap-to-bite scaling parameter, $l(\xi )$, for serological data over a number of herds, was then just the sum of the individual herd marginal log-likelihoods,

5 $$l(\xi ) = \sum_{h}log {L}_{h}\left(\xi \right).$$

The herds we chose to contribute to the log-likelihood were those where BTV was found to be already present at the beginning of the study (see supplementary information for more details), to avoid making further assumptions about the introduction mechanism into the herd.

In practice, the log-likelihood was estimated for a profile of values of $\xi$ by simulating multiple realisations of ${W}_{h}$ for each herd, that is we estimated (4) by Monte Carlo integration for (3) over a range of values of $\xi$, and interpolating between points with polynomial regression. The maximum likelihood estimator, ${\xi }^{*}$, was the maximizer of the marginal log-likelihood function presented in the main text along with confidence intervals derived by a standard comparison to the ${\chi }^{2}$ distribution (see methods section of King et al.^61^ for a brief but comprehensive introduction to maximum likelihood estimation using log-likelihood profiles in the context of inference for dynamical systems).

### Calculating and mapping the herd reproductive ratio for bluetongue

The reproductive ratio for BTV will differ from day to day and across space. This reflects seasonality and variation in both climatic trends, and the population density of midges and livestock hosts. We approached estimating the reproductive ratio for BTV in the spirit of the case reproductive ratio^62^ using a technique already developed for midges spreading BTV^37^. That is, we calculated the expected number of secondary cases amongst hosts due to a host initially infected on each day *t* in each grid cell *x* whilst taking into account how the conditions for BTV transmission at location *x* changed after time *t*, and using the maximum likelihood model for midge biting. The size of each grid cell was determined by the resolution of the relevant climate data. We used the 0.25 degrees longitude and latitude grid resolution of the E-OBS climate datasets to map reproductive ratio estimates for Europe across space and time. Cattle and sheep densities across Europe were drawn from the livestock Geo-Wiki dataset^63^. The livestock Geo-Wiki datasets were more finely resolved (0.0083 degrees grid resolution) than the E-OBS climate datasets. We calculated the reproductive ratio for each grid cell *x* on each day *t* at the resolution of the E-OBS datasets. The cattle and sheep densities for this coarser grained grid were the average densities over the Geo-Wiki cells contained within the coarser grained grid.

The average number of secondary BTV cases amongst all hosts (cattle and sheep) given a host initially infected on day *t* and at grid cell *x*, is denoted ${R}^{(C)}(x,t)$ for an initial infected cow and ${R}^{(S)}(x,t)$ for an initial infected sheep. For both host species, the average number of secondary cases can be calculated by considering; how many days the host's viraemia will last, the rate at which the host is bitten each day, the percentage of the biting midges that will become infected, how many of these biting midges are expected to survive their EIP to become actively infectious, and how many livestock will be successfully infected by those actively infectious midges. The methodology for combining these estimates using information about midge bionomic rates, EIP distribution, and the daily temperatures on each day after the initial host was infected has been developed by Brand and Keeling^37^.

In this study, we adapted the Brand-method for calculating the reproductive ratio to two species, and used the catch-to-biting scalar derived from comparison between the mechanistic transmission model and the herd sentinel serological survey. The cross-transmission between host species depends on how midge bites are distributed between cattle and sheep. We estimated the proportion of midge bites on cattle at grid cell *x*, ${\phi }^{(C)}(x)$, given the availability of sheep using a common relative preference model, e.g. Szmaragd et al.^64^,

6 $${\phi }^{\left(C\right)}\left(x\right)=\frac{{N}^{\left(C\right)}\left(x\right)}{{N}^{\left(C\right)}\left(x\right)+ \pi {N}^{\left(S\right)}\left(x\right)}.$$

where ${N}^{(C)}(x)$ and ${N}^{(S)}(x)$ are, the local density of cattle and sheep at grid cell *x*. Parameter $\pi$ is a measure of the vector preference for sheep compared to cattle; $\pi <1$ indicates preference for cattle, $\pi >1$ preference for sheep. A relative biting study for sheep and cattle has revealed a preference for biting cattle^30^, from which we derived an estimate $\pi =0.115$ for use in this study. We combined ${R}^{(C)}(x,t)$ and ${R}^{(S)}(x,t)$ into a single reproductive ratio by calculating the leading eigenvalue of the next-generation matrix^65^,

7 $$R\left(x,t\right)= \sqrt{{\phi }^{\left(C\right)}\left(x\right){R}^{\left(C\right)}\left(x,t\right)+\left(1-{\phi }^{\left(C\right)}\left(x\right)\right){R}^{\left(S\right)}\left(x,t\right)}.$$

An attractive feature of using the reproductive ratio as a measure of transmission intensity is its uncomplicated relationship with the persistence of transmission; if $R\le 1$ then the infectious pathogen cannot persist. However, we expect that the biting rate, and therefore the reproductive ratio, will vary from herd-to-herd. From Eqs. (1) and (5) we see that the rate of biting from the susceptible midge population at each herd on each day depends on the random coefficients, ${{b}_{l}}^{(h)}$, and daily varying random effects,${{\rho }_{ct}}^{(h)}$ and ${{\varepsilon }_{t}}^{(h)}$,

8 $$\text{Biting rate of susceptible midges per cattle at herd }h\text{ on day }t\propto exp\left({{b}_{l}}^{\left(h\right)}\cdot {Z}_{lt} +{{\rho }_{ct}}^{\left(h\right)} + {{\varepsilon }_{t}}^{\left(h\right)} \right).$$

The daily varying random effects ($\rho$ and $\epsilon$) are averaged over our estimates for ${R}^{(C)}$ and ${R}^{(S)}$ (this can be achieved analytically; see supplementary information for further details), and therefore our estimate of the reproductive ratio does not depend on daily fluctuations in midge activity. However, variation in the herd-grouped random coefficients indicated systematic differences in midge activity between herds that will not ‘average out’ over time. The distribution of ${{b}_{l}}^{(h)}$ therefore implied a distribution of biting rates for herds within each grid cell on each day, and therefore a distribution of values of $R$ for herds in each grid cell and on each day.

We present the distribution of $R$ for herds by considering the reproductive ratio that would be calculated if the random variable in Eq. (8), ${{b}_{l}}^{(h)}\cdot {Z}_{lt}$*,* took its *p*th percentile value every day, denoting this reproductive ratio, ${R}_{p}(x,t)$. ${R}_{p}(x,t)$ estimates the reproductive ratio that *p*% of herd reproductive ratios are *less than* in grid cell *x* on day *t*. Also, we numerically invert the threshold relationship to find the percentage value, ${\varvec{P}}$, such that ${R}_{p}(x,t)$ satisfies the threshold quantity,

9 $${\varvec{P}}\left(x,t\right)=\{1-p \in [\mathrm{0,1}] | {R}_{p}(x,t) = 1 \}.$$

${\varvec{P}}(x,t)$ is therefore an estimate of the percentage of herds that could have a multiplying BTV outbreak in grid cell *x* if BTV was introduced on day *t*.

To enable spatially extending risk predictions for BTV across Europe certain assumptions about how the midge biting model could be interpolated between the trap locations were necessary. The best regression model for midge catches found significantly different seasonality at the Italian catch locations compared to Sweden and The Netherlands. We assumed that day length was a determinant of midge seasonality and overwintering at different latitudes; for example, the first day of the year with a day length shorter than 9 h has been associated with the onset of overwintering in UK midges^66^. We found no midges on days with less than 8.5 h of daylight when trapping, although catching was not attempted outside of March-November so the number of samples was small. Therefore, at latitudes where no day is ever shorter than 8.5 h (lower latitudes than 46^o^N, which is more or less the border of Switzerland and Italy) we used Italian seasonality to predict midge biting. At latitudes that have at least one day shorter than 8 h (higher latitudes than 49^o^N) we used the Swedish and Dutch midge seasonality. Between these two latitudes, a linear interpolation between the predictions of the two seasonal models was applied.

All map images were generated using MATLAB® **contourf** function. Both the livestock density and E-OBS datasets included grid cells that contain only water, these cells were coloured white.

The MATLAB® code for generating the reproductive ratio estimates is available at a github repository (https://github.com/SamuelBrand1/BTV-European-Reproductive-Ratios).

## Supplementary information

Supplementary information 1.

Supplementary information 2.

## Data Availability

The datasets generated and analysed during the current study are available from the corresponding author on reasonable request. Analysis code is available at this github repository. Livestock and climate datasets are publically available as referenced within the paper.

## References

[1] Carpenter S, Groschup MH, Garros C (2013). Culicoides biting midges, arboviruses and public health in Europe. Antiviral Res..

[2] Carpenter S, Veronesi E, Mullens B, Venter G (2015). Vector competence of Culicoides for arboviruses: three major periods of research, their influences on current studies and future directions. Rev. Sci. Tech. Off. Int. Epiz..

[3] Purse BV, Carpenter S, Venter GJ, Bellis G, Mullens BA (2015). Bionomics of temperate and tropical culicoides midges: knowledge gaps and consequences for transmission of culicoides-borne viruses. Ann. Rev. Entomol..

[4] Purse BV (2005). Climate change and the recent emergence of bluetongue in Europe. Nat. Rev. Micro.

[5] Thiry E (2006). Bluetongue in northern Europe. Veter. Rec..

[6] Wilson AJ, Mellor PS (2009). Bluetongue in Europe: past, present and future. Philos. Trans. R. Soc. B Biol. Sci..

[7] Saegerman C, Berkvens D, Mellor PS (2008). Bluetongue epidemiology in the European Union. Emerg. Infect. Dis..

[8] Zientara S, Sánchez-Vizcaíno JM (2013). Control of bluetongue in Europe. Vet. Microbiol..

[9] Sailleau C, Bréard E, Viarouge C (2015). Re-emergence of bluetongue virus serotype 8 in France, 2015. Transb. Emerg. Dis..

[10] Kyriakis CS (2015). Bluetongue in small ruminants: an opinionated review, with a brief appraisal of the 2014 outbreak of the disease in Greece and the south-east Europe. Vet. Microbiol..

[11] Pinior B, Lebl K, Firth C, Rubel F, Fuchs R (2015). Cost analysis of bluetongue virus serotype 8 surveillance and vaccination programmes in Austria from 2005 to 2013. Veter. Rec..

[12] Savini G (2005). Bluetongue virus isolations from midges belonging to the Obsoletus complex (Culicoides, Diptera: Ceratopogonidae) in Italy. Science.

[13] Carpenter S, Szmaragd C, Barber J (2008). An assessment of Culicoides surveillance techniques in northern Europe: have we underestimated a potential bluetongue virus vector?. J. Appl..

[14] De Liberato C (2005). Identification of Culicoides obsoletus (Diptera: Ceratopogonidae) as a vector of bluetongue virus in central Italy. Veter. Rec..

[15] Mellor, P. S. in *Bluetongue viruses* 143–161 (Springer, 1990).

[16] Caracappa S (2003). Identification of a novel bluetongue virus vector species of Culicoides in Sicily. Veter. Rec..

[17] Hoffmann B, Bauer B, Bauer C, Bätza HJ, Beer M (2009). Monitoring of putative vectors of bluetongue virus serotype 8, Germany. . Emerg. Infect..

[18] Cuéllar AC (2018). Spatial and temporal variation in the abundance of Culicoides biting midges (Diptera: Ceratopogonidae) in nine European countries. Parasit. Vect..

[19] Versteirt V, Balenghien T, Tack W, Wint W (2017). A first estimation of Culicoides imicola and Culicoides obsoletus/Culicoides scoticus seasonality and abundance in Europe. EFS3.

[20] Baylis M, Mellor PS, Wittmann EJ, Rogers DJ (2001). Prediction of areas around the Mediterranean at risk of bluetongue by modelling the distribution of its vector using satellite imaging. Veter. Rec..

[21] Calvete C (2009). Ecological correlates of bluetongue virus in Spain: Predicted spatial occurrence and its relationship with the observed abundanceof the potential *Culicoides* spp.. Vector..

[22] Kluiters G (2012). Modelling the spatial distribution of Culicoidesbiting midges at the local scale. J. Appl. Ecol..

[23] Sanders CJ (2011). Influence of season and meteorological parameters on flight activity of Culicoides biting midges. J. Appl. Ecol..

[24] Diarra M (2015). Modelling the abundances of two major culicoides (Diptera: Ceratopogonidae) species in the niayes area of senegal. PLoS ONE.

[25] Searle KR (2012). Identifying environmental drivers of insect phenology across space and time: culicoides in Scotland as a case study. Bull. Entomol. Res..

[26] Ducheyne E (2013). Abundance modelling of invasive and indigenous Culicoides species in Spain. Geospat. Health.

[27] Gubbins S, Carpenter S, Baylis M, Wood JLN, Mellor PS (2008). Assessing the risk of bluetongue to UK livestock: uncertainty and sensitivity analyses of a temperature-dependent model for the basic reproduction number. J. R. Soc. Interface.

[28] Hartemink NA (2009). Mapping the basic reproduction number (R0) for vector-borne diseases: a case study on bluetongue virus. EPIDEM.

[29] Guis H (2011). Modelling the effects of past and future climate on the risk of bluetongue emergence in Europe. J. R. Soc. Interface.

[30] Elbers ARW, Meiswinkel R (2014). Culicoides (Diptera: Ceratopogonidae) host preferences and biting rates in the Netherlands: comparing cattle, sheep and the black-light suction trap. Vet. Parasitol..

[31] Elbers ARW, Meiswinkel R (2015). Limited attractant range of the black-light suction trap for the capture of Culicoidesbiting midges (Diptera: Ceratopogonidae). J. Appl. Entomol..

[32] Koenraadt CJ (2014). Bluetongue, Schmallenberg—what is next? Culicoides-borne viral diseases in the 21st Century. BMC Vet Res.

[33] Mullens BA, McDermott EG, Gerry AC (2015). Progress and knowledge gaps in Culicoides ecology and control. Veterinaria Italiana.

[34] Möhlmann TWR (2018). Community analysis of the abundance and diversity of biting midge species (Diptera: Ceratopogonidae) in three European countries at different latitudes. Parasit. Vect..

[35] Santman-Berends IMGA, Bartels CJM, van Schaik G, Stegeman JA, Vellema P (2010). The increase in seroprevalence of bluetongue virus (BTV) serotype 8 infections and associated risk factors in Dutch dairy herds, in 2007. Vet. Microbiol..

[36] Santman-Berends IMGA, Stegeman JA, Vellema P, van Schaik G (2013). Estimation of the reproduction ratio (R0) of bluetongue based on serological field data and comparison with other BTV transmission models. Preventive Veterinary Medicine..

[37] Brand SPC, Keeling MJ (2017). The impact of temperature changes on vector-borne disease transmission: culicoidesmidges and bluetongue virus. J. R. Soc. Interface.

[38] Calvete C (2008). Modelling the distributions and spatial coincidence of bluetongue vectors Culicoides imicola and the Culicoides obsoletus group throughout the Iberian peninsula. Med. Vet. Entomol..

[39] Calistri P, Goffredo M, Caporale V, Meiswinkel R (2003). The Distribution of Culicoides imicola in Italy: application and evaluation of current mediterranean models based on climate. Zoonoses Public Health.

[40] Pioz M (2011). Estimating front-wave velocity of infectious diseases: a simple, efficient method applied to bluetongue. Veterin. Res..

[41] Pioz M (2012). Why did bluetongue spread the way it did? Environmental factors influencing the velocity of bluetongue virus serotype 8 epizootic wave in France. PLoS ONE.

[42] Nicolas G (2018). Environmental heterogeneity and variations in the velocity of bluetongue virus spread in six European epidemics. Prev. Veter. Med..

[43] Mollison D (1991). Dependence of epidemic and population velocities on basic parameters. Math. Biosci..

[44] Lutscher F (2010). Nonlocal dispersal and averaging in heterogeneous landscapes. Appl. Anal..

[45] Durand B (2010). Anatomy of bluetongue virus serotype 8 epizootic wave, France, 2007–2008. Emerg. Infect. Dis..

[46] Heymann, Y. *CORINE land cover: Technical guide*. (Office for Official Publ. of the Europ. Communities, 1994).

[47] Blackwell A (1997). Diel flight periodicity of the biting midge Culicoides impunctatus and the effects of meteorological conditions. Med. Vet. Entomol..

[48] Vogels CBF (2016). Latitudinal diversity of culex pipiens biotypes and hybrids in farm, peri-urban, and wetland habitats in Europe. PLoS ONE.

[49] Mathieu B (2012). Development and validation of IIKC: an interactive identification key for Culicoides (Diptera: Ceratopogonidae) females from the Western Palaearctic region. Parasit Vect..

[50] Haylock, M. R. *et al.* A European daily high-resolution gridded data set of surface temperature and precipitation for 1950–2006. **113,** D20119–12 (2008).

[51] Bolker BM (2009). Generalized linear mixed models: a practical guide for ecology and evolution. Trends Ecol. Evol..

[52] Stone M (1977). An asymptotic equivalence of choice of model by cross-validation and Akaike's criterion. J. R. Stat. Soc. Ser. B.

[53] Elston DA, Moss R, Boulinier T, Arrowsmith C, Lambin X (2001). Analysis of aggregation, a worked example: numbers of ticks on red grouse chicks. Parasitology.

[54] Turner J (2019). The effect of temperature, farm density and foot-and-mouth disease restrictions on the 2007 UK bluetongue outbreak. Sci. Rep..

[55] Keeling MJ, Rohani P (2008). Modeling Infectious Diseases in Humans and Animals.

[56] Mullens BA, Holbrook FR (1991). Temperature effects on the gonotrophic cycle of Culicoides variipennis (Diptera: Ceratopogonidae). J. Am. Mosq. Control Assoc..

[57] Carpenter S (2011). Temperature dependence of the extrinsic incubation period of orbiviruses in culicoides biting midges. PLoS ONE.

[58] Gerry AC, Mullens BA (2000). Seasonal abundance and survivorship of Culicoides sonorensis (Diptera: Ceratopogonidae) at a southern California dairy, with reference to potential bluetongue virus transmission and persistence. J. Med. Entomol..

[59] Batten CA (2008). Bluetongue virus: European Community inter-laboratory comparison tests to evaluate ELISA and RT-PCR detection methods. Vet. Microbiol..

[60] Davis M (1984). Piecewise-deterministic markov processes: a general class of non-diffusion stochastic models. J. R. Stat. Soc. Ser. B.

[61] King AA, Ionides EL, Pascual M, Bouma MJ (2008). Inapparent infections and cholera dynamics. Nature.

[62] Fraser C (2007). Estimating individual and household reproduction numbers in an emerging epidemic. PLoS ONE.

[63] Robinson TP (2014). Mapping the global distribution of livestock. PLoS ONE.

[64] Szmaragd C (2009). A modeling framework to describe the transmission of bluetongue virus within and between farms in Great Britain. PLoS ONE.

[65] Diekmann O, Heesterbeek J, Metz JA (1990). On the definition and the computation of the basic reproduction ratio R 0 in models for infectious diseases in heterogeneous populations. J. Math. Biol..

[66] Searle KR (2014). Environmental drivers of culicoides phenology: how important is species-specific variation when determining disease policy?. PLoS ONE.

