# Supplementary material for: Biting midge dynamics and bluetongue transmission: a multiscale model linking catch data with climate and disease outbreaks
Source: Sci Rep. 2021 Jan 21;11:1892. doi: 10.1038/s41598-021-81096-9 (PMC7820592; doi:10.1038/s41598-021-81096-9)
Supplement: Supplementary file 1 — Supplementary information 1. [file 41598_2021_81096_MOESM1_ESM.docx]

**Biting midge dynamics and bluetongue transmission: A multiscale model linking catch data with climate and disease outbreaks: Supplementary information**

Tim W. R. Möhlmann^1,2^, Matt J. Keeling^3,4,5^, Uno Wennergren^2^, Guido Favia^6^, Inge Santman-Berends^7^, Willem Takken^1^, Constantianus J. M. Koenraadt^1^, *Samuel P. C. Brand^3,4^

^1^Laboratory of Entomology, Wageningen University and Research Centre, P.O. box 16, 1700 AA, Wageningen, The Netherlands

^2^ IFM Theory and Modelling, Linköping University, 581 83 Linköping, Sweden

^3^School of Life Sciences, University of Warwick, Coventry, UK.

^4^Zeeman Institute, University of Warwick, Coventry, UK.

^5^Mathematics Institute, University of Warwick, Coventry, UK.

^6^School of Biosciences & Veterinary Medicine, University of Camerino, 62032, Camerino, Italy

^7^GD Animal Health, PO Box 9, 7400 AA Deventer, The Netherlands

**Further details on biting midge catch regression**

*Trap latitude-longitude locations:*

|  |  | **Habitat type** | | |
| --- | --- | --- | --- | --- |
|  |  | **Farms** | **Peri-Urban** | **Wetlands** |
| **Country** | **Sweden** | [58.296530,15.584782]  [58.343622,15.602404] [58.330597,15.704327] | [58.416973, 15.499516]  [58.401515, 15.626744]  [58.405494, 15.595035] | [58.362106, 15.651861]  [58.361585, 15.654910]  [58.361542, 15.659072] |
|  | **The Netherlands** | [51.971084, 5.761455]  [51.973637, 5.773978]  [52.013077, 5.645998] | [51.979771,5.660278]  [52.018075,5.655372]  [51.979257,5.645230] | [51.969443,5.758940]  [51.967693, 5.758896]  [51.971671,5.747826] |
|  | **Italy** | [42.914466,13.854588]  [42.944809,13.859857]  [42.943098,13.853856] | [42.883455,13.879388]  [42.951012,13.850783]  [42.934424,13.891933] | [42.896600,13.911895]  [42.899042,13.909813]  [42.903365,13.908667] |

*Full list of variables considered for biting midge catch model fitting:*

There were three sources of climate data considered for catch model regression: 1) **Tinytag® data loggers.** One Tinytag® sensor was used for each habitat/country combination and used to represent the climate at all the trap locations of that habitat/country combination. 2) **Weather stations.** In Sweden and Italy the nearest weather station reporting a time series of climate data was used to represent the climate at all the trap sites in that country. In The Netherlands there were four weather stations approximately equidistant from the trapping locations. The average over all four stations was used to construct the time series of climate data used for all trap sites at the country. 3) **E-OBS** **gridded** **climate data set** (<https://www.ecad.eu> ^1^). We identified the E-OBS grid square containing each trap location, and used the time series of climate variables associated with that grid square.

Following is a summary of variables considered for biting midge catch regression by source, variable type (e.g. temperature, humidity) and time scale (e.g. average over day, week before catches etc) or by variable category and their subdivisions:

**Climate variables:**

- Tinytag® sensor at trap location:
- Hourly temperature (°C)
  - Average over 24 hours before collection
  - Average over last 7 days before collection
  - Average over last 30 days before collection
- Hourly relative humidity (0-100%)
  - Average over 24 hours before collection
- Local weather station data:
- Hourly temperature (°C)
  - Average over 24 hours before collection
  - Average over last 7 days before collection
  - Average over last 30 days before collection
- Hourly relative humidity (0-100%)
  - Average over 24 hours before collection
- Hourly wind velocity (ms^-1^)
  - Average over dawn and dusk before collection
- E-OBS gridded climate data set:
- Daily temperature (°C)
- Daily precipitation (mm)
  - Average over last 7 days before collection
  - Average over last 30 days before collection
  - Average 8-30 days before collection
  - Coefficient of variation over last 30 days (std. dev. of daily precipitation divided by mean)
- Daily air pressure
  - - Day of collection
  - Yearly periodic sine and cosine functions
  - Bi-annually periodic sine and cosine functions
  - Tri-annually periodic sine and cosine functions

**Categorical variables:**

- Number of hours of daylight exceeding a threshold
- 10 hours of daylight as threshold
- 9 hours of daylight as threshold
- Country
- Sweden/The Netherlands vs Italy
- Each country
- Habitat
- Farm habitat vs not farm habitat
- Each habitat type

**Random effect grouping variables only:**

- Unique code for each collection (this models overdispersion in catch sizes relative to Poisson model).
- Unique variable for each day of collection and country pair (this models autocorrelation between catches collected on the same day in the same country).

We also considered product effects of climate variables with specific habitat types to account for some climate variables being important predictors for only particualr habitat types.

*Details of best fit GLMM model for biting midge catch size:*

The target for regression was the aggregate number of Obsoletus group*, C. chiopterus,* Pulicaris group *and C. punctatus* midges captured over 24 hours. As well as identifying potential predictor variables (see above), we also performed GLMM model selection. As described in the main text we used backwards model selection ^2^ to eliminate potential predictor variables from GLMM models containing large numbers of variables by considering improvement in AICc. The full saturated model contained too many variables to be fitted by the MATLAB® **fitglme** function (the design matrix for the saturated model is not of full rank), and returned an error. Therefore, we used a selection of highly over parameterised models. The presented best fit model was selected each time by the backwards model selection.

We present the best fit model variable coefficients along with standard errors, a t-statistic for the null hypothesis that the variable coefficient is zero and the p-value implied by this t-statistic (degrees of freedom were 427; Table S1). We also present the estimated covariance matrix of the multivariate normally distributed location grouped random effect variables and estimated variances of the normally distributed autocorrelation and overdispersion effects (Table S2). Finally, the improvement in AICc for the best fit model compared to some selected alternative models are shown (Table S3). All GLMM regression was performed using the MATLAB® **fitglme** function, with the setting that corresponds to the Laplace approximation approach to estimating fixed effect coefficients and the variance structure of the random effects.

**Table S1. Fixed effect regression coefficients for biting midge abundance model.**

| Fixed Effects | | | |
| --- | --- | --- | --- |
| **Variable (dataset)** | **Estimate (SE)** | **T-statistic** | **P-value** |
| Climatic variables | | | |
| Mean daily temperature 24hrs before collection (E-OBS) | 1.2687 (0.2304) | 5.5057 | 6.3574e-08 |
| Squared mean daily temperature 24hrs before collection (E-OBS) | -0.0292 (0.007401) | -3.9434 | 9.3884e-05 |
| Mean daily precipitation 7 days before collection (E-OBS) | 0.2391 (0.06895) | 3.4682 | 0.0005771 |
| Precipitation CoV 30 days before collection; **wetlands only** (E-OBS) | 1.0584 (0.1543) | 6.8572 | 2.472e-11 |
| Mean daily wind velocity 24hrs before collection (local weather stations). | -0.2692 (0.13154) | -2.0467 | 0.04130 |
| Habitat variables | | | |
| Farm environment (Italy) | 8.2523 (0.8906) | 9.2658 | 9.6007e-19 |
| Farm environment (Sweden and The Netherlands) | 4.0519 (0.9078) | 4.4634 | 4.9258e-06 |
| Seasonality variables (Italy) | | | |
| Intercept | -16.3872(2.0078) | -8.1617 | 3.7455e-15 |
| Annual cosine | 1.9527 (0.6205) | 3.1471 | 0.0017645 |
| Annual sine | 0.8710 (0.2397) | 3.6336 | 0.00031352 |
| Seasonality variables (Sweden and The Netherlands) | | | |
| Intercept | -11.5931 (1.9051) | -6.0853 | 1.8919e-07 |
| Annual cosine | 4.1756 (1.1193 ) | 3.7305 | 0.04768 |
| Annual sine | -0.9064 (0.3411) | -2.6573 | 0.00031352 |
| Bi-annual cosine | 2.105 (0.72941) | 2.8859 | 0.0041003 |
| Bi-annual sine | -0.6901 (0.24171) | -2.8551 | 0.0045102 |
| Tri-annual cosine | 1.9411 (0.45136) | 4.3006 | 2.1138e-05 |

**Table S2. Random effects regression covariance matrix for three levels: Location specific effects, autocorrelation and remaining unexplained overdispersion.**

| Random effect group | Covariance matrix | | |
| --- | --- | --- | --- |
| Trapping Location |  | | |
|  | **Intercept** | **Mean daily temperature** | **Mean daily wind velocity** |
| **Intercept** | 1.6226 | -0.1105 | -0.0385 |
| **Mean daily temperature** | -0.1105 | 0.0223 | -0.1444 |
| **Mean daily wind velocity** | -0.0385 | -0.1444 | 0.1138 |
| Autocorrelation |  |  |  |
|  | **Intercept** |  |  |
| **Intercept** | 0.7540 |  |  |
| Overdispersion |  |  |  |
|  | **Intercept** |  |  |
| **Intercept** | 1.3479 |  |  |

| Alternate regression model | $\Delta$AICc |
| --- | --- |
| CoV of daily precipitation as a predictor for all habitat types, not just wetland. | 24.9 |
| Wetland and peri-urban habitats as distinct effects | 2.2 |
| One seasonal component for all countries | 49.1 |
| Different seasonal component for each country | 4.6 |
| Independent location-specific random effects | 11.6 |
| No location level random effects | 75 |

**Table S3. Change in AICc from the best fit model for some selected variant models.**

*The choice of temperature variable on predictive model*

The three sources of temperature data used as potential predictor variables for the midge capture regression model were highly correlated (Fig. S1). Therefore, model reduction using AICc to compare models selected only one temperature data source, since the other two add no extra information. For this regression model the favoured temperature data set was from the E-OBS climate dataset, which was surprising because this was an average over a coarse-grained cell (0.25 degree resolution), whereas the TinyTag and weather station data were more local to the trapping site. However, the statistical support for the regression model used in this paper was marginal, since the difference in AICc between a regression model using either of the other two temperature datasets was small; ΔAICc = 0.07 for using TinyTag temperature data and ΔAICc = 0.03 for using weather station data. This reflected that using any of the temperature data sources gave very similar results.

|  |
| --- |
| **Figure S1**. Correlation between temperature data sources for each trapping day. *Left:* Comparison between the TinyTag temperature sensor left nearby a trapping site and the E-OBS temperature for the cell containing the trapping location. *Right:* Comparison between the weather station temperature data left nearest to the trapping site and the E-OBS temperature for the cell containing the trapping location. |

**Mechanistic transmission model details and likelihood of herd serological data**

In this section, we expand upon the description of the mechanistic model for herd-level transmission in the main text.

*Transmission model at herd level:*

We infer a proportionality factor between the biting rate of midges on cattle and the number of midges we would expect to catch over 24 hours, using the sentinel cattle prevalence data as data for the inference. To do this we construct a mechanistic model, which predicts the dynamics of cattle BTV prevalence given:

1. the temperature dependent rates of individual biting midge bionomics and virology (biting rate, incubation rate, and mortality rate) drawn from the literature,
2. the statistical model for 24 hours of biting midge catches (which depends on temperature and other climatic variables) reinterpreted as being proportional to the biting rate per cattle per day from susceptible midges,
3. and the proportionality factor $\xi$ (the target parameter for inference) between the catch model and the daily bites on cattle.

For each herd we introduce state variables: numbers of susceptible cattle ($S_{C}$), infectious cattle in their *n*th viraemic stage ($I_{C}^{(n)}$), recovered/immune cattle ($R_{C}$), latent infected midges in their *m*th extrinsic incubation period (EIP) phase ($E_{M}^{(m)}$), and infectious midges ($I_{M}$) (see Fig. 6 in main text for a schematic diagram). The dynamics of BTV transmission in each herd follows deterministic per-capita rates determined by the relevant transmission and recovery events:

- Susceptible cattle are infected, becoming infectious, at a rate proportional to the number of infectious midges, the daily biting rate per midge ($\alpha$), and the probability of transmission to cattle per bite $P_{MC}$.
- Infectious cattle remain infectious for a gamma-distributed period with mean duration $1/\gamma$, which corresponds to sub-dividing the cattle infectious period into $n_{I}$ discrete compartments each with mean duration ${1/\gamma n}_{I}$.
- Once an infectious cattle completes all its infectious periods it remains immune to re-infection with BTV.
- Each cattle is bitten by susceptible midges at a rate $\xi B$ where $B$ is the expected number of midges that would have been caught according to the statistical trapping model and the proportionality factor $\xi$ is to be inferred. Therefore, the rate at which new midges are infected is proportional to; $\xi B$, the number of infectious cattle, and the probability of transmission to susceptible midges per bite ($P_{CM}$). Note that this modelling choice implies that the number of susceptible midges biting hosts is not depleted, which is valid when the number of infected midges is small compared to the total population size.
- Infected midges are not immediately infectious. Latent infected midges become infectious after a (gamma distributed) EIP, which corresponds to a $n_{E}$ sub-divided period, each sub-division being completed at a rate $\nu n_{E}.$ After becoming infectious, midges remain infectious for the rest of their lives.
- Midges die at a rate $\mu$. Note that not all midges will survive their EIP.

We denote the time series of climate variables selected for the best fit regression model (see above) $\mathcal{C(}t)$. Midge bionomic processes are temperature dependent, therefore the atmospheric temperature influences both the rate of susceptible midges arriving to bite cattle, and the rate at which midges which have become infected with BTV incubate the virus experience mortality and make post-infection bites. The temperature at time *t* is denoted $\mathcal{T(}t)$. The time variable *t* is continuous, however we treat climate variables as being constant over each day and changing as a daily varying step function. Exact herd locations were redacted. However their containing zone was available for each herd (see ^3^ for further details). The daily values of $\mathcal{C(}t)$ and $\mathcal{T(}t)$ for a herd were the spatial average for that day, over all E-OBS grid cells coinciding with, or contained within, the control zone containing the herd.

Wind velocity is a factor in the best fit regression model, but we found it significantly harder to access detailed spatio-temporal data about wind velocities across Europe. The mean daily wind velocity (at dawn and dusk) during the period of midge catch collections was 3.22ms^-1^. We used this as a point estimate for each day at each herd, which after fitting the proportionality factor $\xi$ effectively corresponds to increasing the variance of the predictions for biting midge catches (since wind velocity was also a location group random variable).

The best fit biting midge catch regression model includes random effects grouped at the level of location and day of collection. This means that some of the random effects are associated with the herd location, while others are associated with the day. These random effects represent unobserved causes of variation in the midge catch. The location group random effects at herd *h* were modelled as drawn from a distribution of values,$b_{h}\sim Normal(0,\Sigma)$, where the covariance matrix $\Sigma$ is given above (Table S2). Note that the location group random effects are only drawn once per simulation since they are associated with the location and do not change in time. The autocorrelation ($\rho_{ct}$) and overdispersion effects ($\varepsilon_{ht}$) vary daily and are redrawn for each day, as independent normally distributed random variables with mean zero and variance as given above (Table S2). From equation (1) in the main text, the rate of biting from the susceptible midge population at herd *h* on each day *t* is conditional on the random effects;

| $B\left( t \right)= \xi\exp( \beta\cdot X_{ht} +b_{h}\cdot Z_{ht} +\rho_{ct} + \varepsilon_{ht})$ | (S1) |
| --- | --- |

Where the predictor variables for the fixed effects ($X_{ht}$) and random effects ($Z_{ht}$) can be derived each day from the climate time series for the herd, as well as the day of year to include seasonality in midge activity. Here $\beta$ denotes the fixed effect coefficients for the best fit model (Table S1). Equation (S1) expands equation (2) in the main text.

The description above can be summarised as a random ODE model ^4^ (because of the random effects) for transmission at each herd:

|  | (S2) |
| --- | --- |

$\alpha(\mathcal{T),}\nu(\mathcal{T),}\mu(\mathcal{T)}$ are the midge biting, incubation, and mortality rates expressed as functions of temperature (see Table S4 for functions and parameter estimates). Apart from modelling biting from the susceptible midge population as a random process proportional to the prediction of a GLMM fitted to midge catch data, the model described is identical to Gubbins *et al*.^5^. Simulations of this model were performed in MATLAB® using the **ode45** solver function.

*Likelihood function for seroprevalence data from The Netherlands sentinel survey*

In the main text, we described how repeated simulation was used to marginalise over the random effects and estimate the likelihood function for $\xi$. We now give further details. The 2007 serological surveillance study in The Netherlands for sentinel cattle herds is fully described in Santman-Berends *et al*. ^3,6^. In summary, 270 herds were recruited into the study from 20 control zones covering the entirety of The Netherlands. Each herd had an initial survey of more than 26 cattle (exact numbers varied). The survey occurred in June 2007 (exact initial survey day varied). In some herds there were already seropositive cattle in the initial survey, however in each case at least 16 initially seronegative cattle were select for future testing. Each month from July to December 2007, the herds were revisited and 16 cattle from the initially seronegative group were selected to test for development of seropositivity. Sensitivity and specificity of the ELISA test for BTV is high ^7^, therefore we simplified our likelihood calculations by treating the test as perfect.

As described in the main text Methods we map the timing of ELISA detectability (8-9 days after infection and for a long period after the end of viraemia ^7^) onto our multi-compartment transmission model: cattle in the first two stages of the multi-stage cattle BTV-infectiousness period, that is on average the first 8.2 days of infectiousness, have not developed sufficient antibodies to be ELISA detectable. Whereas cattle in subsequent stages of infectiousness were detectable by milk ELISA. Furthermore, we assumed that the study duration was short enough that no cattle in the recovered stage had lost sufficient antibodies to become undetectable.

We initialised simulations for each herd by assuming that the proportion of cattle found to be seropositive in the initial survey at each herd represented the true frequency of seropositivity in the herd. We lacked data on whether the cattle confirmed as seropositive were still infectious. We therefore made the maximum entropy choice that the seropositive cattle were distributed evenly between the ELISA detectable model compartments ($I_{C}^{(3)},I_{C}^{(4)},I_{C}^{(5)}, R_{C})$. It was highly probable that some cattle that were reported as seronegative were infectious but not detectable. Therefore, we also assumed that there were initially as many cattle in each ELISA undetectable model compartment as each ELISA detectable model compartment. These assumptions implied that for each herd the initial values of detectable model compartments obeyed:

| $I_{C}^{(3)}(0)+I_{C}^{(4)}(0)+I_{C}^{(5)}(0)+R_{C}(0) = {N\cdot SP}_{t_{0}}$  $I_{C}^{(1)}(0)=I_{C}^{(2)}(0)=I_{C}^{(3)}(0)=I_{C}^{(4)}(0)=I_{C}^{(5)}(0)=R_{C}(0)$ | (S3) |
| --- | --- |

Where ${SP}_{t_{0}}$ is the proportion of cattle found seropositive in the initial survey of the herd (on day $t_{0}$) and $N$ is the size of the herd.

In 2007 BTV spread across The Netherlands, broadly travelling from south to north. We avoided making additional assumptions about the mechanism of introduction into herds by restricting our analysis to herds in the south of the country (control zones 15-20 in Santman-Berends *et al*. ^3,6^), where BTV seropositive cattle were found in the initial herd survey (this reduced the number of herds in the data set from 270 to 60). We also assumed that the number of infected midges present at the beginning of the simulation and arriving over the simulated period were negligible compared to the number infected midges generated by the initially infected cattle (that is every midge compartment was initially zero).

Apart from being identified as initially seronegative, the cattle that was tested each month after the initial survey, were not separated from the rest of the herd. Therefore, they are as likely to become infected with BTV as other cattle. For any set of random effects (the full set of random effects for herd *h* is denoted $W_{h}$; see main text), we can numerically solve the transmission model (S2) for the proportion of cattle who have become seropositive by time *t* amongst the cattle which were found to be initially seronegative:

| $P(t;W_{h},\xi) = \frac{I_{C}^{(3)}(t;W_{h},\xi)+I_{C}^{(4)}(t;W_{h},\xi)+I_{C}^{(5)}(t;W_{h},\xi)+R_{C}(t;W_{h},\xi) - {N\cdot SP}_{t_{0}}}{N - {N\cdot SP}_{0}}.$ | (S4) |
| --- | --- |

In which we have explicitly included the dependence on ${(W}_{h},\xi)$ in the solution of model (S2). Care was taken to match the simulation time *t* to calendar time so that the climate each herd experienced in the simulation matched the data on real climatic conditions in 2007.

The likelihood of the testing data for herd *h*, for a particular set of random effects ($W_{h}$), and a given value of the scale factor $\xi$, is the product over binomial probabilities. The likelihood contribution for the i^th^ test on the herd *h* of detecting $n_{i}$ seropositive cattle from the $N_{test,i}$ tested cattle on testing day *t_i_*, is the binomial probability of the test. The total likelihood contribution of the herd *h* is:

| $L_{h}(W_{h},\xi) =\prod_{i} \mathcal{B}in(n_{i}; N_{test,i}, P(t_{i};W_{h},\xi)).$ | (S5) |
| --- | --- |

Equation (S5) corresponds to equation (2) in the main text.

As described in the main text the marginal likelihood for $\xi$ without random effects was found by repeatedly simulating model (S2) and using that,

| $\frac{1}{n}\sum_{k=1}^{n} L_{h}(W_{h}^{(k)},\xi) \to_{n\to\infty}\int L_{h}(w,\xi)f(w) \text{d}w = L_{h}(\xi).$ | (S6) |
| --- | --- |

Where $W_{h}^{(k)}$ is the *k^th^* independent realisation of the random effects for herd *h* drawn from the density function $f(w)$, which encodes the distribution described by Table S3. For each herd we used n=1000 simulations to marginalise over the random effects for a range $\xi= 0.01, 0.02, ... , 1.5$. As described in the main text we constructed a full marginalised log-likelihood for each of these values of $\xi$ by adding the log-likelihoods for each herd. This gave a noisy estimate of the log-likelihood profile $l(\xi)$, which we smoothed using a fourth order polynomial. From the smoothed log-likelihood profile we derived the maximum likelihood estimator $\xi^{*} = 0.53$ given in the main text. A 95% confidence region $CI(\xi) = [0.40, 0.68]$ was also derived as the region of $\xi$ values with log-likelihood 'close' to the maximum log-likelihood,

| $CI(\xi) = \left\{ \xi\vert2[l(\xi^{*}) - l(\xi)] < c \right\}$ | (S7) |
| --- | --- |

The 'closeness' threshold $c$ is defined by $\mathbb{P(}\chi_{1}^{2} < c) = 0.95$, this comparison between log-likelihood profile and a chi-squared random variable (with 1 degree of freedom) is a standard method for constructing confidence regions (see King *et al*. for further description in the context of inference for dynamical systems ^8^).

| 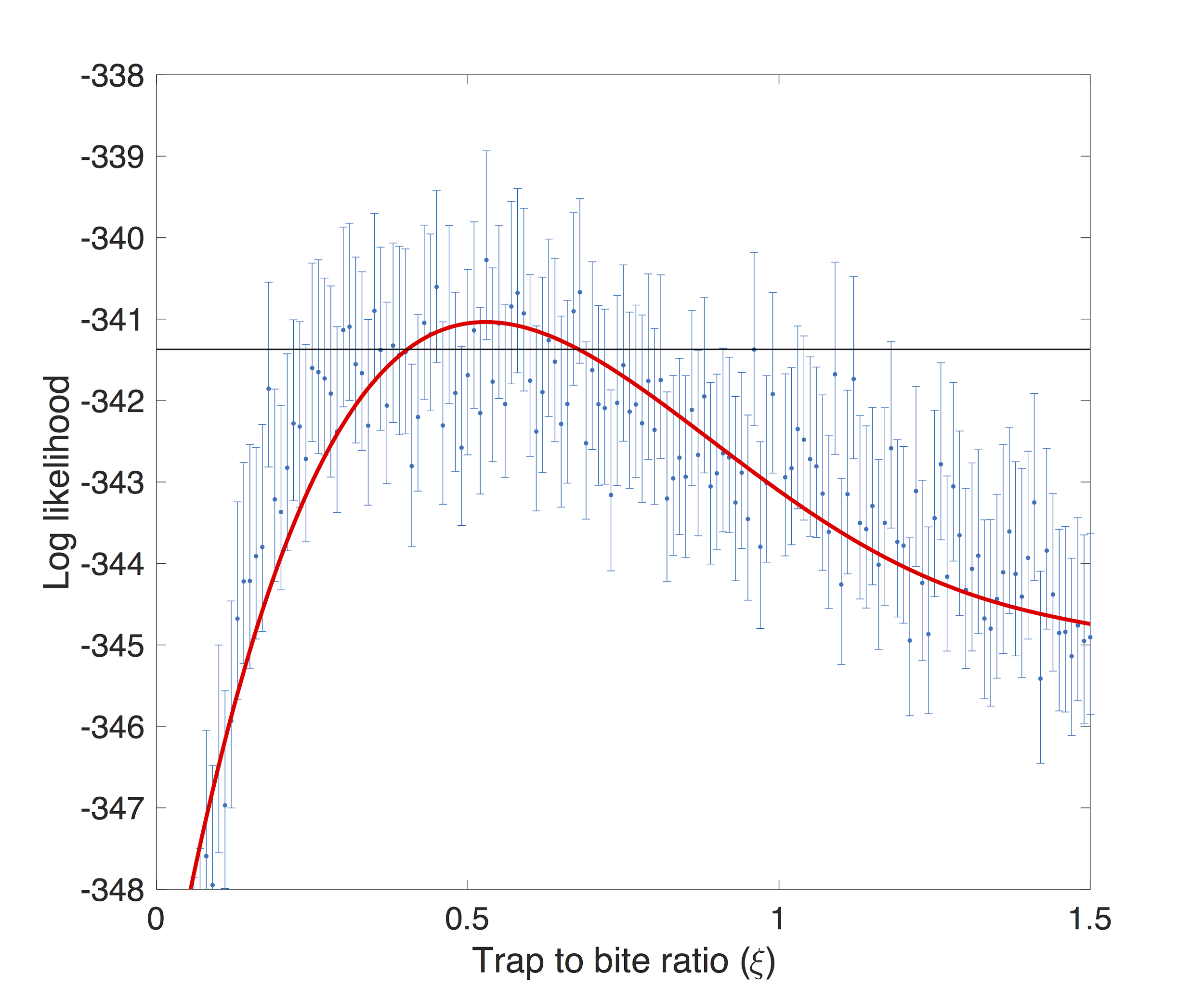 |
| --- |
| **Figure S2**. The log-likelihood profile for the trap to bite ratio scalar ($\xi$). Blue dots and error bars represent the log-likelihood estimate and uncertainty for each value of $\xi$. The red curve gives a fourth order polynomial smoothing of the noisy log-likelihood estimates from which we calculate a maximum likelihood estimate and 95% confidence region (values of $\xi$above the black line). |

*Estimates for epidemiological parameters for herd transmission model*

**Table S4. Parameters for herd level transmission model. Estimate range for temperature dependent rates are their 10^o^C - 30^o^C values**

| **Description** | **Symbol** | **Estimate** | **Comments** | **References** |
| --- | --- | --- | --- | --- |
| Probability of transmission from vector to host | $P_{MC}$ | 0.9 | Point estimate within range. | ^5,9^ |
| Probability of transmission from host to vector | $P_{CM}$ | 0.05 | Point estimate within range. | ^10^ |
| Midge biting rate | $\alpha\boldsymbol{(}\mathcal{T}\boldsymbol{)}$ | 0.05-0.40 (day^-1^) | Biting rate depends on temperature $\mathcal{T}$:  $\alpha(\mathcal{T) =}\frac{\mathcal{T(T-}3.7)(41.9-\mathcal{T})^{0.37}}{5000}$ | ^11^ |
| Midge mortality rate | $\mu\boldsymbol{(}\mathcal{T}\boldsymbol{)}$ | 0.05-1.1 (day^-1^) | Mortality rate depends on temperature $\mathcal{T}$:  $\mu\left( \mathcal{T} \right)=0.009\exp\left( 0.16\mathcal{T} \right)$ | ^12^ |
| Extrinsic incubation period  - number of Erlang stages | $n_{E}$ | 10 | Point estimate within range. | ^13^ |
| Extrinsic incubation period  - incubation rate | $\nu(\mathcal{T)}$ | 0-0.3 (day^-1^) | Incubation rate depends on temperature $\mathcal{T}$:  $\nu(\mathcal{T}) =0.018(T-13.4)$ | ^13^ |
| Viraemic duration of cattle  - number of Erlang stages | $n_{I}$ | 5 | From viraemic duration data fitted to an Erlang (Gamma) distribution. | ^14,15^ |
| Viraemic duration of cattle  - recovery rate | $\gamma$ | $1/20.6$ (day^-1^) | From viraemic duration data fitted to an Erlang (Gamma) distribution. | ^14,15^ |

*Additional information on spatially varying reproductive ratio calculations*

In this section we give additional information on the calculation method used for spatially varying reproductive ratios presented in the main text. We concentrate on the differences between the method used in this paper and the approach used in Brand and Keeling ^16^. In this work we consider sheep and cattle, use a regression model for biting derived for this paper, and include the effect of sheep mortality on the estimate of the reproductive ratio.

The idea is to calculate the case reproductive ratio $R(t)$ for BTV in the sense of Fraser ^17^, which calculates the expected number of secondary cases generated by an initial host infected at time *t*. Thereby taking into account that the conditions for transmission might change with time. This approach is computationally challenging because it requires not only information about the climate drivers for BTV at time *t* but also all times $\tau$ *> t*, noting that midges infected by the host will persist after the end of the individual’s infectious period. This approach to calculating $R(t)$ for BTV is fundamentally different from constructing $R$ as a function of temperature (e.g. ^5,18^) which implicitly assumes that temperature will remain constant over the host’s infectious period and the remaining lifetimes of the infected midges which survive the end of the host’s infectious period.

In Brand and Keeling ^16^ a per-vector capacity $\hat{C}(t)$ was defined as the expected number of successful infections due to a single midge alive on day *t*, and calculated using direct summation over expected outcomes on every day $\tau$ *> t* (see equation (2.5) in Brand and Keeling). The per-vector capacity could be defined either as “alive at the beginning of day *t*” (as in Brand and Keeling) or “alive at end of day *t* having bitten an infected host”. The ratio between the two definitions is the probability of the midge biting an infected host and surviving the day. The first definition is more appropriate when considering a fixed sized midge population, the second definition we used for this model since, by assumption, $B(t)$ is our estimate of the number midges biting per cattle on day *t*. The expression $B(t)$ hides the dependence on random effect coefficients (equation (S1)). Since the autocorrelation and overdispersion effects vary daily, it is natural to average out their effects using,

| $\mathbb{E}\left[ \text{exp}\left( \rho_{ct} + \varepsilon_{ht} \right) \right]=\text{exp([}\sigma_{\rho}^{2}\text{+}\sigma_{\varepsilon}^{2}\text{]/2).}$ | (S8) |
| --- | --- |

Where $\sigma_{\rho}^{2}$ and $\sigma_{\varepsilon}^{2}$ are the variances of the autocorrelation and overdispersion effects estimated during the biting midge catch regression (see Table S2 for estimates). We could also average over the location-group random effects, which would lead to an estimate of the reproductive ratio for a ‘typical’ herd. However, we were more interested in estimating the proportion of farms with reproductive ratio greater than unity across time and space in Europe. Therefore, we used as our estimates of daily bites per animal:

| $B_{p}^{\left( C \right)}\left( t \right)=\xi^{*}exp( \beta\cdot X_{ht} +{[b_{h}\cdot Z_{ht}\text{]}}_{p}\text{ + (}\sigma_{\rho}^{2}\text{+}\sigma_{\varepsilon}^{2}\text{)/2),}$  $B_{p}^{(S)}\left( t \right)={\pi\xi}^{*}\exp( \beta\cdot X_{ht} +{[b_{h}\cdot Z_{ht}\text{]}}_{p}\text{ + (}\sigma_{\rho}^{2}\text{+}\sigma_{\varepsilon}^{2}\text{)/2).}$ | (S9) |
| --- | --- |

Where $\pi=0.115$ is an estimate of the relative preference of biting sheep rather than cattle (see main text), and ${[b_{h}\cdot Z_{ht}\text{]}}_{p}$ is the p percentile value of the random variable $b_{h}\cdot Z_{ht}$. Following Brand and Keeling this gives species-specific reproductive ratios at (approximately) p percentile for herds as:

| $R_{p}^{\left( C \right)}\left( t \right)=\sum_{\tau=t}^{\infty} B_{p}^{\left( C \right)}\left( \tau\right)\hat{C}(t)P_{H}^{\left( C \right)}(\tau,t)\text{,}$  $R_{p}^{\left( S \right)}\left( t \right)=\sum_{\tau=t}^{\infty} B_{p}^{\left( S \right)}\left( \tau\right)\hat{C}(t)P_{H}^{\left( S \right)}(\tau,t)\text{.}$ | (S10) |
| --- | --- |

Where $P_{H}^{\left( C/S \right)}(\tau,t)$ are the probabilities that a cattle/sheep infected on day *t* is still infectious during day $\tau>t$. The infectious duration of cattle is treated as a five stage Erlang process averaging 20.6 days (see above), the time taken to complete a *n*-stage, mean $\mu$ Erlang process (*T*) is known to be $T \sim\text{Gamma(}n,n/\mu$). Therefore $P_{H}^{\left( C \right)}\left( \tau,t \right)\mathbb{= P(}T^{(C)}> \tau-t)$ uses the upper tail function of a $T^{(C)}\text{ \textasciitilde Gamma(}5,5/20.6)$ random variable. Note that we are assuming that mortality has a negligible effect on cattle, since BTV disease induced mortality was found to be low for cattle during the post-2006 BTV-8 epidemic in northern Europe ^19^. For sheep excess mortality due to BTV infection is not ignored, we use a point estimate for the per capita disease induced excess mortality rate amongst sheep of $d=$0.0055 (day^-1^) within the range of published mortality estimates ^15,19^. The distribution of infectious period for sheep we use has mean 16.4 days: $T^{(S)}\text{ \textasciitilde Gamma(}14,14/16.4)$ ^15,20^. Both death and the end of BTV infectiousness stop midge biting on sheep, therefore,$P_{H}^{\left( S \right)}\left( \tau,t \right)\mathbb{= P(}T^{\left( S \right)}> \tau-t)\text{exp(-d(}\tau-t)\text{)}$. The factor $\text{exp(-d(}\tau-t)\text{)}$ is the chance that the infected sheep is still alive $\tau-t$ days after infection. The $R_{p}(x,t)$ values used in the main text to construct estimates of the percentage of farms in grid cell *x* that have a reproductive ratio greater than unity on day *t* were all derived by applying equation (S10) to the main text equation (7).

**References for supplementary information**

1. Haylock, M. R. *et al.* A European daily high-resolution gridded data set of surface temperature and precipitation for 1950–2006. *J Geophys Res* **113,** D20119–12 (2008).

2. Bolker, B. M. *et al.* Generalized linear mixed models: a practical guide for ecology and evolution. *Trends Ecol Evol* **24,** 127–135 (2009).

3. Santman-Berends, I. M. G. A., Stegeman, J. A., Vellema, P. & van Schaik, G. Estimation of the reproduction ratio (R0) of bluetongue based on serological field data and comparison with other BTV transmission models. *Prev Vet Med* **108,** 276–284 (2013).

4. Han, X. & Kloeden, P. E. *Random Ordinary Differential Equations and Their Numerical Solution*. (Springer, 2017).

5. Gubbins, S., Carpenter, S., Baylis, M., Wood, J. L. N. & Mellor, P. S. Assessing the risk of bluetongue to UK livestock: uncertainty and sensitivity analyses of a temperature-dependent model for the basic reproduction number. *J R Soc Interface* **5,** 363–371 (2008).

6. Santman-Berends, I. M. G. A., Bartels, C. J. M., van Schaik, G., Stegeman, J. A. & Vellema, P. The increase in seroprevalence of bluetongue virus (BTV) serotype 8 infections and associated risk factors in Dutch dairy herds, in 2007. *Vet Microbiol* **142,** 268–275 (2010).

7. Batten, C. A. *et al.* Bluetongue virus: European Community inter-laboratory comparison tests to evaluate ELISA and RT-PCR detection methods. *Vet Microbiol* **129,** 80–88 (2008).

8. King, A. A., Ionides, E. L., Pascual, M. & Bouma, M. J. Inapparent infections and cholera dynamics. *Nature* **454,** 877–880 (2008).

9. Baylis, M., O’Connell, L. & Mellor, P. S. Rates of bluetongue virus transmission between *Culicoides sonorensis* and sheep. *Med Vet Entomol* **22,** 228–237 (2008).

10. Carpenter, S. & Mordue, W. Oogenesis and laboratory survival in the Scottish biting midge *Culicoides impunctatus*. *Physiol Entomol* (2006). doi:10.1111/j.1365-3032.2005.00478.x

11. Mullens, B. A., Gerry, A. C., Lysyk, T. J. & Schmidtmann, E. T. Environmental effects on vector competence and virogenesis of bluetongue virus in *Culicoides*: interpreting laboratory data in a field context. *Vet Ital* **40,** 160–166 (2004).

12. Gerry, A. C. & Mullens, B. A. Seasonal abundance and survivorship of *Culicoides sonorensis* (Diptera: Ceratopogonidae) at a southern California dairy, with reference to potential bluetongue virus transmission and persistence. *J Med Entomol* **37,** 675–688 (2000).

13. Carpenter, S. *et al.* Temperature dependence of the extrinsic incubation period of Orbiviruses in *Culicoides* biting midges. *PLoS One* **6,** e27987 (2011).

14. Melville, L. F. *et al.* in *Bluetongue Disease in southeast Asia and the Pacific* (eds. St George, T. & Kegao, P.) 245–250 (ACIAR Proceedings series, 1996).

15. Szmaragd, C. *et al.* A modeling framework to describe the transmission of bluetongue virus within and between farms in Great Britain. *PLoS One* **4,** e7741 (2009).

16. Brand, S. P. C. & Keeling, M. J. The impact of temperature changes on vector-borne disease transmission: *Culicoides* midges and bluetongue virus. *J R Soc Interface* **14,** 20160481–13 (2017).

17. Fraser, C. Estimating individual and household reproduction numbers in an emerging epidemic. *PLoS One* **2,** 758 (2007).

18. Turner, J., Bowers, R. G. & Baylis, M. Two-host, two-vector basic reproduction ratio (R0) for bluetongue. *PLoS One* **8,** e53128 (2013).

19. Elbers, A. *et al.* Field observations during the bluetongue serotype 8 epidemic in 2006: I. Detection of first outbreaks and clinical signs in sheep and cattle in Belgium, France and the Netherlands. *Prev Vet Med* **87,** 21–30 (2008).

20. Veronesi, E., Hamblin, C. & Mellor, P. S. Live attenuated bluetongue vaccine viruses in Dorset Poll sheep, before and after passage in vector midges (Diptera: Ceratopogonidae). *Vaccine* **23,** 5509–5516 (2005).
